# Pore Network Percolation in Polymers of Intrinsic Microporosity Examined by Gaseous and Antimatter Probes

**DOI:** 10.1021/acs.macromol.5c00945

**Published:** 2025-10-09

**Authors:** Darin Sukalingum, Tianran Zhai, Marc H. Weber, Sohraab A. Khan, Thant H. Htut, Jeremy I. Feldblyum

**Affiliations:** † Department of Chemistry, 1084 University of Albany, State University of New York, 1220 Washington Avenue, Albany, New York 12226, United States; ‡ Department of Chemistry, University of Albany, State University of New York, 1220 Washington Avenue, Albany, New York 12226, United States; § Institute of Materials Research, Washington State University, Pullman, Washington 99164, United States

## Abstract

Synthesis of polymers with tailored properties requires an understanding of the underlying phenomena leading to these properties. Polymers of intrinsic microporosity (PIMs) are non-network polymers with a substantial guest-accessible free volume. Their BET surface areas have been reported to be as high as 1000 m^2^/g, and their porosity has led to the demonstration of some of the best membrane-based gas separations to date. However, it has been challenging to predict porosity in PIMs a priori, in part due to an incomplete framework for understanding the origins of porosity in these materials. Here, porosity in several archetypal PIMs is examined through the lens of a percolation model, whereby porosity in oligomers follows behavior expected for nonpercolated and percolated networks. Positron-based Doppler broadening spectroscopy is used to examine buried pores in samples whose pores are inaccessible to typical (nonantimatter) guests and demonstrate that buried pores are present in PIMs with nonpercolated pore networks. Taken together, the findings are in agreement with the contention that network percolation plays a critical role in pore accessibility in PIMs.

## Graphical abstract

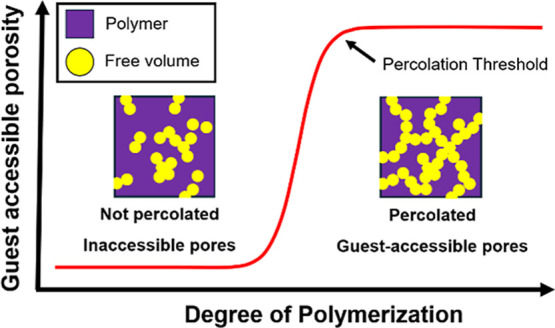

## Introduction

Designing polymers with carefully tailored properties remains a great challenge due to the often-substantial deviation between predicted and realized sample characteristics.^1^ Nonuniform molecular mass distributions, impurities introduced during synthesis, multiple termination pathways, and other irregularities all can contribute to synthetic outcomes potentially differing from expectation. Non-network microporous polymers (polymers containing <2 nm vacancies or pores), termed polymers of intrinsic microporosity (PIMs),^2,3^ offer no exception. These polymers are an emerging class of materials that demonstrate promise for catalysis,^4^ sensing,^5^ guest uptake/capture,^6^ separation,^7–10^ energy production/storage,^11^ and other applications.^3^ These polymers’ versatility relies not only on their physical and chemical robustness but also on their varied pore characteristics (e.g., size, shape, and chemistry).^4,9,12^

The permanent porosity of PIMs is currently understood to result from some combination of interrelated factors including backbone rigidity, backbone contortions, limited molecular mobility, and inefficient packing.^2,13–18^ However, the extent to which these or other factors contribute to pore network formation and guest-accessible porosity in these materials remains unestablished, further complicating efforts to rationally design PIMs with matching synthetic outcomes.

The emergence of porosity in other porous media such as porous silica and carbons has been rationalized using percolation theory.^19–23^ Instances of percolation theory in the polymer literature are not uncommon as this theory has been used to rationalize many polymer properties exhibiting “all or none” behavior (i.e., the conductivity of polymer composites).^24–35^ However, while the pore networks of PIMs have been presumed to be interconnected,^36,37^ percolation models have yet to be assessed as descriptors for how porosity can emerge (or remain unachieved) in this class of materials. In this work, we show that the percolation theory provides a sound framework for understanding the emergence of porosity in PIMs.

Percolation theory describes the behavior of networks on an infinite lattice with *N* number of sites as constituents are added or removed.^38^ The occupancy of any site in the lattice is statistical (with probability *p*) and independent of the occupancy of its neighbors. Hence, the population of such sites is given by the *pN*. When *p* = 0, all sites are unoccupied. As *p* increases, the site occupancy increases, leading to the eventuality of lattice occupants having neighbors. Groups of contiguous lattice occupants are termed clusters. As *p* further increases, clusters begin to grow, and eventually a lattice occupant in one cluster will have an occupant in another cluster as its neighbor, thus merging the two clusters into one.

Cluster formation, expansion, and merging continue until a critical *p* is reached (denoted as *p*_*c*_), after which a slight increase in *p*(*N*) leads to most, if not all, clusters merging into one unified cluster (i.e., forming a percolated network). This network constitutes a contiguous path from one end of the lattice to the opposite end. Such a cluster is termed an infinite spanning cluster.^24,38,39^ The critical probability of site occupancy (*p*_*c*_) follows the principles of Kolmogorov’s zero-one law,^40^ which states that the likelihood of a given event occurring is either 0 or 1. With respect to percolation theory, if *p* < *p*_*c*_, the probability of the existence of an infinite spanning cluster is zero, and if *p* > *p*_*c*_, the probability is one.^38^ In order to switch between *p* < *p*_*c*_ and *p* > *p*_*c*_, a region of rapid rate of change must connect the two states, which gives rise to the characteristic sigmoidal trend associated with percolation theory.

To assess the extent to which percolation theory might describe porosity in PIMs, we sought to vary the pore number density (*p*, in the context of percolation theory) in a series of model polymers. It is well known that as the value of the number-average molecular weight (*M*_n_) increases for a polymer, its properties bear a diminishing resemblance to those of the monomer. For example, solubility typically decreases with increasing *M*_n_.^41^ In addition, the wavelength of π–π* transitions in conjugated polymers shifts to longer wavelengths as *M*_n_ increases due to lower-energy requirements for electronic excitation.^42,43^ While a relationship between *M*_n_ and porosity has not been established for PIMs, Smith and co-workers recently demonstrated a positive correlation between the side chain length and porosity in bottlebrush polymers.^44,45^ Hence, we hypothesized that the porosity/pore number density might be controlled through the PIM backbone chain length. Should percolation play a role in pore network formation, a sigmoidal increase in the BET surface area (one measure of porosity^46^) is expected as chain lengths are increased.

## Results and Discussion

The model polymers MPIM-1, PIM-1, and TBPIM-9 (Scheme 1) were selected for this study. These polymers are not susceptible to aging under moderate thermal treatment or time scales. In addition, this selection includes structurally distinct motifs that are responsible for engendering microporosity. The chemistries of these polymers also differ; both purely organic (PIM-1 and TBPIM-9) and metal-containing (MPIM-1) polymers were selected. The variety of chosen polymers enables the assessment of the proposed theory over a range of structurally distinct PIMs. MPIM-1 was developed by our group in 2021 ^15^ and features backbone ferrocene, which introduces contortions and thus inefficient intra- and interchain packing. BET surface areas of this polymer were reported to reach 400 m^2^/g. PIM-1, one among the first set of PIMs reported, was described by Budd et al. in 2004.^16^ This polymer possesses spiro-centers that inhibit efficient packing and a BET surface area of 850 m^2^/g.^2,17^ Finally, TBPIM-9 takes advantage of Tröger’s base to twist the polymer backbone. This polymer was developed by Carta et al. in 2014 and has a BET surface area of 680 m^2^/g.^18^ Intrinsic microporosity in these polymers stems from the highly rigid Tröger’s base-fused ring systems that link monomer units and restrict conformational freedom.^18^

**Scheme 1:**
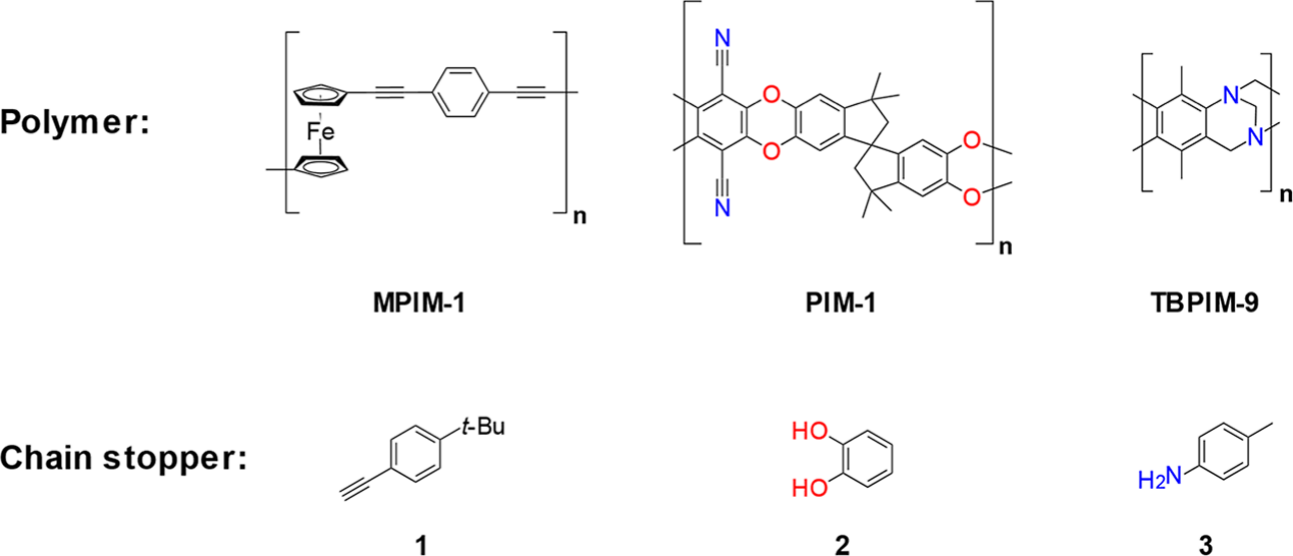
Chemical Structure of Polymers (Top Row) Utilized in This Investigation and the Respective Chain Stopper Employed in Their Polymerization (Bottom Row)^a^

a 1:4-*tert*-Butylphenylacteylene, 2: catechol, 3: *p*-toluidine.

To achieve control over the polymer chain length, chain stoppers were used (Scheme 1). This strategy uses monofunctional monomers (chain stoppers) to terminate PIM step growth; increasing the chain stopper concentration reduces the average chain length according to the Carothers equation^47^ (see Supporting Information for further details). The use of chain stoppers is a well-known method for synthesizing short oligomers with a specific average chain length.^43,48–51^

After preparation and activation (see full details in the Experimental Section and Supporting Information), the BET surface areas of MPIM-1, PIM-1, TBPIM-9, and their oligomers were determined. The BET surface area vs calculated chain length for MPIM-1, PIM-1, and TBPIM-9 are presented in Figure 1.

**Figure 1:**
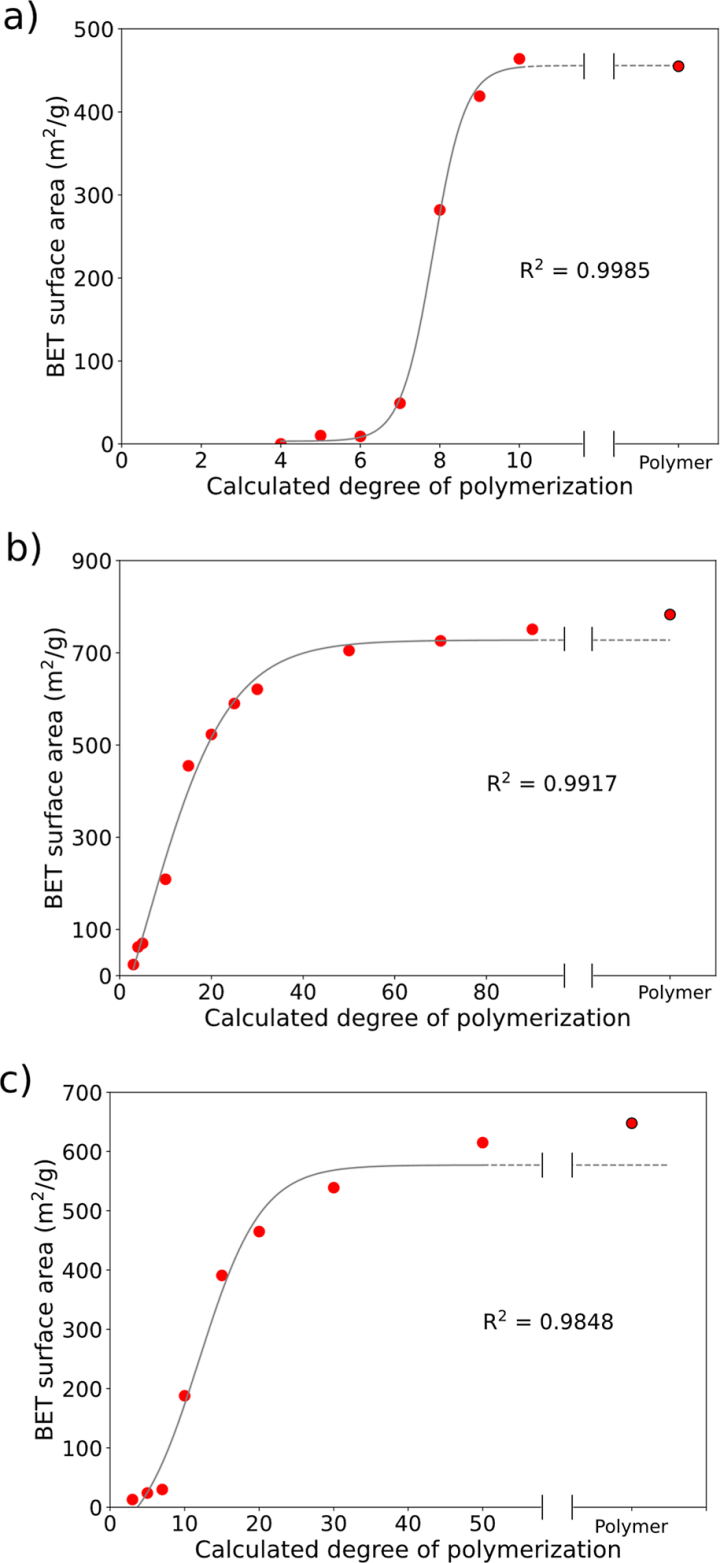
BET surface area vs calculated degree of polymerization (with Sigmoidal–Boltzmann fits) for (a) MPIM-1, (b) PIM-1, and (c) TBPIM-9. Red ● Experimental data, — Sigmoidal–Boltzmann model, - - - Sigmoidal–Boltzmann model (extrapolated).

The data for oligomeric MPIM-1 exhibit three notable regions (Figure 1a). The 4-mer to 6-mer region is characterized by relatively low and similar surface areas. The 6-mer to 9-mer region exhibits a sharp increase (>400 m^2^/g) in the BET surface area over small changes in chain lengths. Finally, chains with an average calculated degree of polymerization above 9 show high surface areas with negligible differences as the chain length is increased.

The plot associated with PIM-1 (Figure 1b) possesses two of the three regions found for MPIM-1. A sharp increase in the BET surface area is present from the 3-mer to 30-mer region, analogous to the 6-mer to 9-mer region of MPIM-1 oligomers. For PIM-1, the increase in the surface area was larger (>600 m^2^/g). Above 30 repeat units, changes in the surface area level off (albeit with a modest increase of 162 m^2^/g from the 30-mer to the polymer). The BET surface area of TBPIM-9 followed similar trends (Figure 1c). As chain lengths are increased, there are regions of low and negligibly changing surface areas (3-mer to 7-mer), sharply increasing (>500 m^2^/g) surface area (7-mer to 30-mer), and high but negligibly changing surface area (30-mer to polymer).

The data comparing the oligomer length and BET surface area are well fit to a Sigmoidal–Boltzmann model^52–54^ (see the Experimental Section), in agreement with the contention that percolation theory plays a key role in free volume network formation in porous polymers. At the smallest chain lengths, it is possible that the pore population grows as the degree of polymerization is increased. However, if most of these pores are inaccessible to external guests, they will not contribute to the measured BET surface area. Pore population growth can also lead to the pores merging into clusters. Once a threshold pore population is approached, inaccessible clusters begin merging with guest-accessible clusters, leading to the formation of larger continuous, accessible pore networks and thus sharp increases in the surface area over small increases in chain lengths. Beyond the percolation threshold, a single continuous network that encapsulates most of the material’s pores is formed. Hence, increasing the chain length past this point does not substantially increase the BET surface area, as pore network formation is largely complete.

The sigmoidal relationships between the chain length and BET surface area found for the PIMs studied herein are consistent with the contention that network percolation is achieved after the threshold chain lengths. Should network percolation indeed play a primary role in the emergence of porosity in PIMs, we contend that (1) complete network percolation is achieved at chain lengths beyond the percolation threshold and (2) the presence of inaccessible pores and pore clusters for PIMs of chain length below the percolation threshold. We examine these contentions below.

To assess the presence of fully percolated networks in PIMs of chain lengths greater than the percolation threshold, the BET surface areas of oligomer mixtures were measured (preparation details for the oligomer mixtures are supplied in the Supporting Information). Specifically, oligomers of PIM-1 with chain lengths calculated to be 90 and 5 repeat units were utilized. The 90-mer exhibits surface areas close to those of the polymer and is of length beyond the critical percolation threshold (Figure 1b). Hence, these oligomers are hypothesized to possess fully percolated networks. The 5-mer, having a low surface area (38 m^2^/g), is below the percolation threshold and is presumed to lack a fully percolated internal pore network. In a physical mixture of 5-mer and 90-mer, it is expected that the 90-mer will make a positive contribution to the surface area of the mixture, while the 5-mer’s contribution will be negligible. Therefore, as the 5-mer content in the mixture is increased, the surface area is expected to decrease linearly. If the mixture was instead dissolved and reprecipitated (solution blend; see Supporting Information), differing outcomes are expected, depending on whether a percolated network is or is not present in the 90-mer. Should the 90-mer possess a percolated pore network that makes major contributions to its porosity, the short chains of the 5-mer might obstruct the pore network formed in the larger oligomer, rendering parts of the percolated network inaccessible to external guests. Such obstruction would lead to a sharp decline in the BET surface area, resulting in a deviation from the surface areas of physical mixtures. If the 90-mer’s high porosity is not the result of a percolated pore system, the surface areas of the solution blends are expected to follow a similar trend to that of the physical mixtures, as there would be no percolated network to obstruct.

As expected, increasing the 5-mer content of the physical mixture leads to a linear reduction in the BET surface area (Figure 2). However, in stark contrast, as the 5-mer content is increased in the solution blend above 40 wt %, the BET surface area undergoes a sharp, nonlinear decline, consistent with crossing of a percolation threshold. This observation is in agreement with the contention that accessible porosity is derived from percolated pore networks in PIMs.

**Figure 2:**
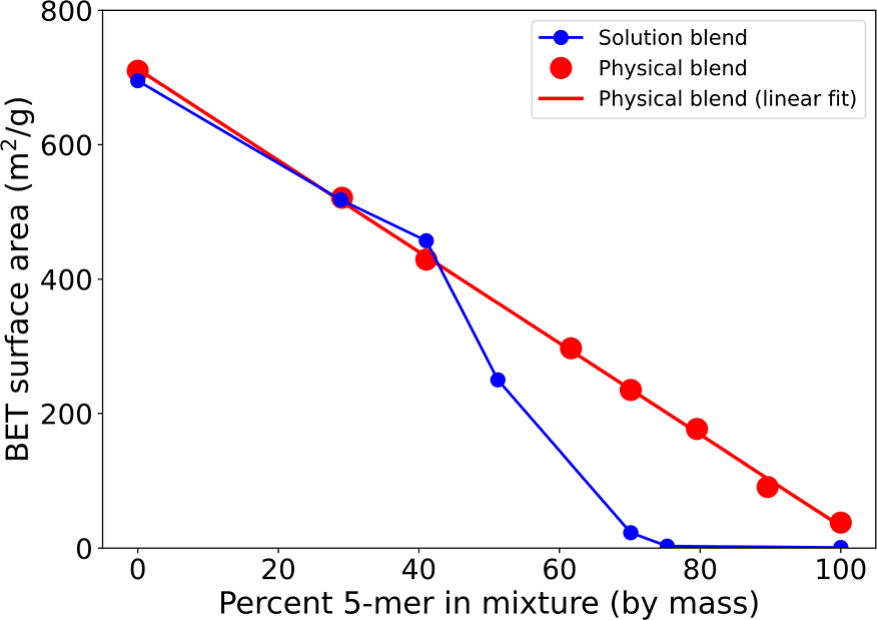
BET surface area vs mass percent 5-mer in 90-mer/5-mer mixture of PIM-1. Physical blending refers to mixing the oligomer powders. Solution blending refers to dissolution and reprecipitation of the physical mixtures.

These results also support the contention that the reduction in the BET surface area can be rationalized by considering pore blocking in an initially percolated network. The pore networks of the 90-mer would be expected to possess many free volume paths to the internal pore network. At relatively low but increasing wt % 5-mer in the solution-blended mixtures, surface area declines with the increasing nonporous content, similar to the trend observed for the physical mixtures. The effects from pore blocking are limited at this stage as multiple paths of access to the internal pore network still remain, even if some of those paths become obstructed. At a threshold wt % 5-mer in the mixtures, access to the internal pore networks becomes restricted. As such, small increases in 5-mer loading in the mixtures past this threshold can render sections of the internal network completely inaccessible, leading to sharper losses in the surface area compared to what are observed for the physical blended mixtures (where internal pore networks remain accessible). This steep drop-off in surface area continues with increasing 5-mer loading until the percolated network is completely obstructed, rendering the mixture nonporous.

Nonpercolated pore networks in PIMs are presumed to possess inaccessible pores/pore clusters. However, techniques such as gas sorption analysis are unable to probe these potential pores, as they are not guest-accessible. Doppler broadening spectroscopy (DBS) overcomes this barrier by utilizing positrons as the probe. Positrons can penetrate matter and return information pertaining to packing defects and pores in the material. DBS measures the energy shift of positron annihilation products (gamma photons) relative to those of an electron–positron system at rest.^55,56^ The annihilation product for thermalized *para*-positronium (an electron–positron-bound pair with antiparallel spins) is two 511 keV gamma photons 180° apart. If the pair is not at rest, the resulting angle between the annihilation photons shifts from 180° in order to conserve momentum.^55,56^

The deviation of the angle between the annihilation products directly impacts the shift in gamma photon energy according to the equation

$${\Delta E}_{\gamma }=mcv_{cm}\cos\;\text{ϕ}$$

where Δ*E*_γ_ is the shift in the energy of the 511 keV line, *m* is the mass of the bound pair, *c* is the speed of light, *v*_*cm*_ is the speed of the center of mass of the bound pair, and *ϕ* is the angle between the direction of the center of mass and one of the emitted gamma photons.^55^ Before annihilation, most positrons tend to approach or attain thermal equilibrium while traveling through matter; hence, the predominant contributions to the momentum of the positron–electron pair are from the electron.^55–57^ Two important variables that can be derived from DBS studies are the shape (S) parameter and the wing (W) parameter. Relatively high S parameter and low W parameter values are the result of positron annihilations with low momentum valence or free electrons, which are indicative of defects or vacancies within the studied material. Low S parameter and high W parameter values (the result of positrons annihilating with high momentum core electrons) can be interpreted as a relatively lower presence of defects and vacancies within the studied material.^56–58^ Here, DBS analysis was carried out on the 5-mer (nonporous) and 10-mer (410 m^2^/g) of MPIM-1 as a function of depth from the oligomer surface (see Experimental Section).

Differences between the surface layers (0–1.27 μm) and bulk regions of the oligomers are immediately apparent. The 10-mer possesses a relatively higher free volume content near the surface compared to the 5-mer based on the high S parameter and low W parameter of the 10-mer relative to those of the 5-mer. However, the bulk region (1.3–30 μm) of both materials reveals near-identical annihilation properties (similar S and W parameter values; the highlighted region of Figure 3). The bulk regions of both oligomers having similar annihilation properties can be interpreted as the two materials having similar free volume characteristics. This finding stands in contrast to the results of gas sorption analysis, where the 10-mer exhibits guest-accessible porosity, but the 5-mer does not. Together, the results from gas sorption and DBS show that while the 5-mer does possess internal free volume, that volume is inaccessible to external guests. These results are in perfect agreement with the contention that inaccessible pore/pore clusters are present in PIM oligomers, an outcome expected for nonpercolated networks.

**Figure 3:**
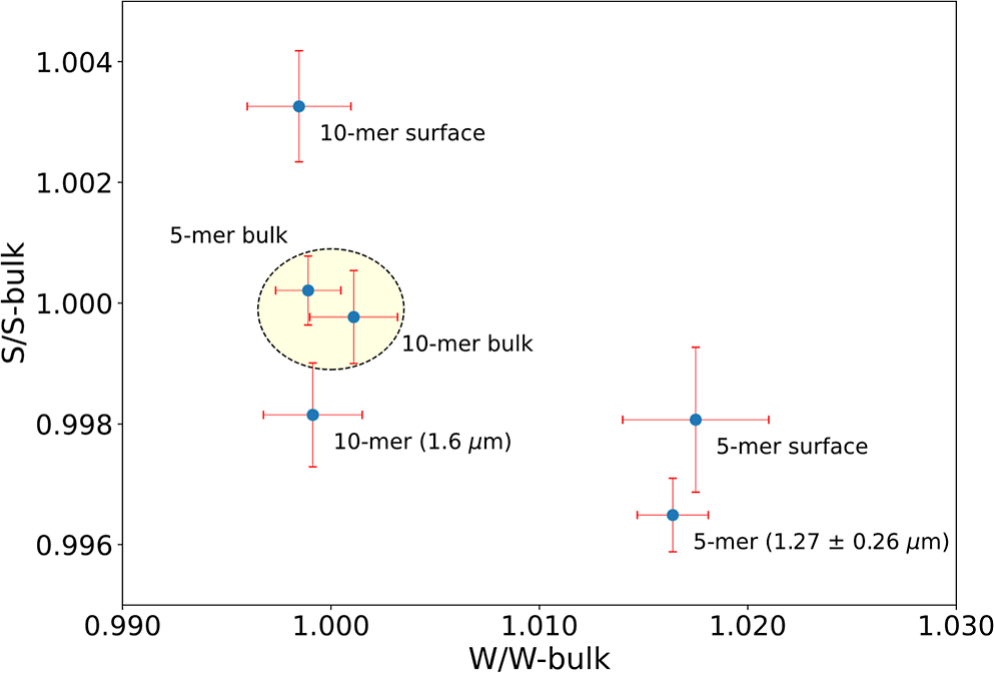
S-parameter vs W-parameter evaluation for the 5-mer and 10-mer of MPIM-1 at various penetration depths. The S- and W-parameters for the bulk region of both oligomers are statistically similar (highlighted region).

## Conclusion

This investigation aimed to assess whether percolation theory provides an appropriate model for understanding how porosity emerges in PIM oligomers. The growth of fully percolated networks in PIM-1, MPIM-1, and TBPIM-9 oligomers was investigated by measuring the porosity as a function of chain length in these oligomers. A sigmoidal relationship between the BET surface area and oligomer chain length was observed, a hallmark of pore network percolation and an indication that percolation theory plays a key role in pore network formation.

Percolation in PIM oligomers was assessed in addition in two ways. First, physical vs solution blending of short and long oligomers reveals that smaller oligomers can act as obstructing agents for the pore networks of the larger oligomers. The observed sharp decline in surface area for solution-blended mixtures, absent in physical mixtures, reflects the presence of a percolated network that is disrupted on the addition of a threshold amount of a pore-blocking agent. Second, DBS analysis of longer porous and shorter nonporous MPIM-1 oligomers reveals that the free volume distribution in the bulk region of the oligomers is near-identical. This finding shows that, despite being nonporous by gas sorption analysis, shorter oligomers do possess nonpercolated pores.

The findings herein contribute to our understanding of the origins of the porosity in PIMs. They suggest that eliminating small molecular mass impurities (including unreacted monomers and shorter oligomers) could enable network percolation in PIMs that would otherwise lack guest-accessible pores.

## Experimental Section

### Sample Preparation for N_2_ Isotherm Acquisition

All samples to be used for N_2_ isotherm data collection were either oven-dried (100 °C for PIM-1 samples) or air-dried (for MPIM-1 and TBPIM-9 samples) prior to activation. PIM-1 polymer and oligomers were activated for >20 h at reduced pressure and constant temperature (120 °C, <50 mTorr). MPIM-1 polymer and oligomers were activated for >24 h, also at room temperature and reduced pressure (ca. 22 °C, <50 mTorr). TBPIM-9 polymer and oligomers were activated for >18 h at reduced pressure and constant temperature (120 °C, <50 mTorr). After activation, samples were immediately affixed to the gas sorption analyzer (3Flex, Micromeritics Inc., Norcross, GA) for data collection at 77 K. Data points were then collected between *P*/*P*_0_ = 0.01 and 0.9 to construct the adsorption and desorption isotherms.

A 2022 study demonstrated that there are issues involved with the reproducibility of the BET surface area values, which arise from the utilization of differing Rouquerol criteria between users (user bias) during data analysis.^59^ To overcome this obstacle, Osterrieth et al. developed an analysis program titled BET Surface Area Identification (abbreviated BETSI), which uses its own Rouquerol criteria, to make an unambiguous BET surface area evaluation based on the provided isotherm data.^59^ Throughout this study, all BET surface area values were obtained using the BETSI program to reduce user bias during the evaluation.

### Gel-Permeation Chromatography Analysis

The GPC-SEC instrument (EcoSEC High Temperature GPC System with RI detector, Tosoh Bioscience LLC., San Francisco, CA) was set to run using tetrahydrofuran (THF) at 40 °C, and it was therefore a requirement that samples were soluble in THF. To prepare a sample, no more than 1 mg of analyte was added to a clean vial and then dissolved in 5 mL of THF (HPLC grade only). The solution was then taken up in a syringe and transferred to a GPC-compatible vial by passing the solution through a 0.22 μm syringe filter. The prepared samples were then transferred to the instrument for analysis. All calibrations were performed by using a polystyrene standard in THF.

### Doppler Broadening Spectroscopy

Sample selection: for this analysis, we sought a PIM having both nonporous and porous oligomers below and immediately above the percolation threshold, respectively. The 5- and 10-mers of MPIM-1 satisfy this criterion; the other PIMs examined in this manuscript lack definitively nonporous oligomers below the percolation threshold (see Figure 1).

Second, although MPIM-1 possesses metal centers in its ferrocene units and metals are known to inhibit or quench positronium formation, ferrocene itself presents a unique exception. A study carried out by Marques Netto and co-workers demonstrates that positronium formation can be observed in pure ferrocene, unlike in other metallocenes (such as cobaltocene and nickelocene).^60^ This unique exception stems from the electronic structure of ferrocene (which adopts a noble gas configuration according to MO theory), which avoids the positronium inhibiting/quenching abilities typical of iron cations in other contexts.^60^ As such, it is possible to analyze MPIM-1 using positronium-based techniques, even though the polymer contains metal centers.

Sample mounting: for the positron measurements, the polymer powders were pressed onto a carbon tape, similar to a tape used for electron microscopy. A blank tape reference was used to ensure that no signal stemmed from the tape. The powder grains are on the order of 100 μm in diameter, which is sufficiently large to prevent practically all positrons from reaching grain boundaries, except at very low beam energies when the positron may diffuse back to the macroscopic surface of the sample powder. The powder-coated tape is mounted on a vertical sample holder in the vacuum system and evacuated to better than 2.0 × 10^–6^ Torr.

Positron annihilation Doppler broadening measurements were carried out at the Washington State University beam. Positrons from a ^22^Na isotope source are moderated with a thin W foil, accelerated to a beam energy from 0 to 70 keV, and magnetically guided to the samples for depth-resolved measurements. Positrons thermalize within 5 ps and diffuse through the material until trapping at open volume spaces and eventually annihilate with electrons. The incident positron energy can be converted to the mean implantation depth *d* following the empirical formula d·ρ = 40 nm·g·cm^–3^·(E/keV)^1.6^. Here, a density of 1 g/cm^3^ was used (or the product of depth and density).

The two-photon annihilation line–shape parameters S and W are determined by the fraction of two-photon annihilations of the total peak events with a detected energy in a narrow window at the center of the annihilation line and symmetric windows at the edges of the annihilation line, respectively (see the annihilation line in Figures S11 and S12 of the Supporting Information). The window for S is 1.47 keV wide, and each wing window is 3.21 keV wide, starting at 2.57 keV from the center in both directions. The high-efficiency high-purity germanium detector has an energy resolution (full width at half-maximum) of 1.56 keV. At each mean implantation depth, about 1–2 million events are accumulated in the 2-photon annihilation line.

Three-photon annihilations result in a broad triangular energy distribution that peaks at 511 keV. In the window from above the Compton edge to the onset of the two-photon peak, only three photon events are recorded (in addition to background). This allows for the study of an additional parameter called the R parameter, which entails the fraction of events in this window relative to the total in the 2-photon peak. R is sensitive to three-photon annihilations that occur at the surface of the sample and when positrons are trapped at a larger porosity with pore sizes in excess of about 1 nm. The R-parameter measures the combined result of 3-photon events increasing with pore size and concentration.

In both of the studied samples, a 3-photon signal was observed at the surface in addition to a component due to near-surface porosity (Figure 4). It should be noted that the high 3-to-2-photon ratio at the surface is likely due to the backscatter/diffusion of *o*-Ps and its annihilation in vacuum predominantly through a 3-photon decay. The initial steep decrease in R from the surface to about 25 nm is due to surface properties. Below that, the 10-mer sample contains a thin, roughly 0.2 μm thick layer with pores. The 5-mer sample has a much smaller subsurface porosity signal. As such, the increase in R from 5- to 10-mer may stem from larger pores or a higher pore concentration near the surface of the sample.

**Figure 4:**
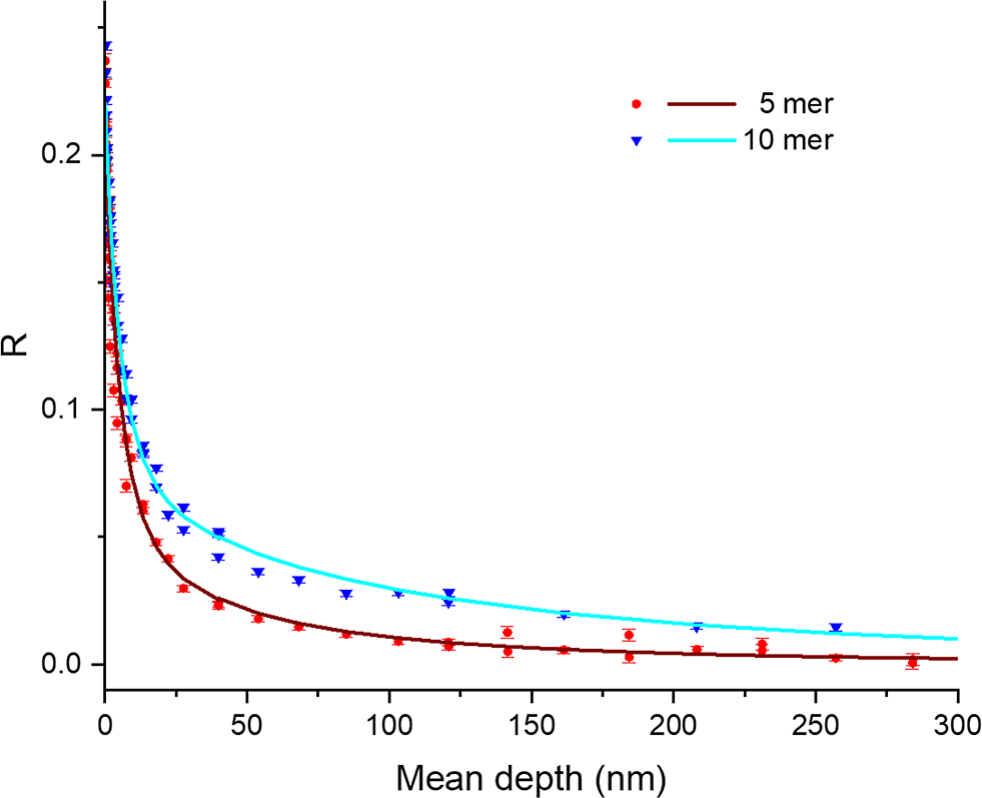
R-parameter signal vs mean positron implantation depth below the surface. The density is assumed to be 1 g/cm^3^.

Additional details on the experimental methodology and sample preparation are supplied in the Supporting Information.

### ^1^H NMR Spectra Acquisition

Approximately 1 mg of analyte was dissolved in 1.0 mL of deuterated chloroform (CDCl_3_). The sample was then transferred to a clean and dry NMR tube, and the tube was capped to prevent solvent evaporation. The tube was then affixed to the instrument for analysis. Number of scans/acquisitions per spectrum = 64.

A Bruker Avance 500 MHz NMR spectrometer (Bruker Scientific LLC, Billerica, MA) was used for all data acquisition.

### Direct Analysis in Real-Time Mass Spectrometry

No special sample preparation is required for this analysis. Analytical conditions: temperature = 400 °C, orifice 1 (20 V, 120 °C), orifice 2 (5 V), ring lens (5 V), ion guide RF (600 V), detector (2000 V), and ion mode (positive). Polyethylene glycol (PEG) was used as the calibration standard.

An AccuTOF-DART-4G Mass spectrometer (JEOL USA, Inc., Peabody, MA) was used for all data acquisition.

### Python Fitting Algorithm

The fitting algorithm employed was created using Python and employs a scientific computation library (Scientific Python or SciPy) to fit the data with the Sigmoidal–Boltzmann model as well as return a coefficient of correlation (*R* ^2^) value for the fit.

The Sigmoidal–Boltzmann model was employed for data fitting in this study, as it allowed for manipulation of the lower bounds of the sigmoidal curve, which is usually set to zero in other models.^52–54^ At low *M*_n_, BET surface area values may not necessarily be at zero, and as such, sigmoidal models that possess a lower bound fixed at zero may produce poor fits to data, even if a clear sigmoidal trend exists.

### Synthesis of Monomers, Oligomers, and Polymers

All reagents were purchased and used without further purification unless specified otherwise.

#### 1, 1′-Diiodoferrocene

A clean and flame-dried 3-neck 1000 mL round-bottom flask was charged with a stir bar, ferrocene (10 g, 53.8 mmol), anhydrous and degassed hexane (180 mL), tetramethylethylenediamine (20 mL, 134.4 mmol), and 2.5 M *n*-butyllithium (*n*-BuLi) in hexane (53.6 mL, 134.4 mmol) under argon protection. The reaction mixture was then allowed to stir for 20 h at room temperature, after which a copious amount of orange precipitate was observed in the flask. While still stirring under argon protection, the flask was then cooled to −78 °C in a dry ice/acetone bath. The flask was then charged with anhydrous and degassed tetrahydrofuran (400 mL), followed by freshly crushed iodine (30 g, 118.2 mmol). The contents were stirred for 15 min at −78 °C. The dry ice/acetone bath was then removed, and the contents were allowed to slowly warm up to room temperature while still stirring for 2 h. The contents of the flask became very dark in color as the contents warmed up and the reaction proceeded.

After 2 h, the reaction was terminated through the slow addition of methanol (20 mL) and stirred for 15 min. The volatiles were removed, and the resulting dark oil was reconstituted with hexane. Two layers were observed: an upper hexane layer and a lower dark oily layer. The hexane layer was collected, and the lower oily layer was washed with hexane (3 × 200 mL). The organic material was then washed with saturated sodium thiosulfate (400 mL) and dried over anhydrous sodium sulfate.

The volatiles were removed once again, and the resulting red oil was subjected to sublimation under reduced pressure (ca. 159 °C, 10 Torr) and then vacuum distillation (ca. 159 °C, 10 Torr) for 1 h each so as to remove most of the residual ferrocene and iodoferrocene. The remaining oil in the still flask was then reconstituted with hexane, and the organics were washed with 0.5 M aqueous iron­(III) chloride (6 × 200 mL), followed by water (3 × 200 mL). The organics were then dried over anhydrous sodium sulfate. The dried organics were then cooled to −78 °C in a dry ice/acetone bath to afford orange needle-like crystals (6.9490 g, 15.8725 mmol). The crystals were collected via vacuum filtration on a prechilled sintered funnel and washed with a minimal amount of hexane prechilled to −78 °C. The filtrate was recooled to −78 °C to afford a second batch of needle-like crystals (2.7620 g, 6.3088 mmol). Crystals were collected by vacuum filtration and washed with a minimal amount of prechilled hexane. The total yield of 1, 1′-diiodoferrocene was 9.7110 g (22.1814 mmol, 41%). It should be noted that the crystalline product has a low melting point (room temperature) and is, therefore, stored at −20 °C. 1, 1′-Diiodoferrocene: ^1^H NMR (CDCl_3_, 500 MHz): δ_H_ 4.18 (t, 4H), 4.37 (t, 4H). *m*/*z*: 437.8 (FcI_2_^+^), 311.9 (FcI^+^).

#### MPIM-1 and Its Oligomers

A clean 50 mL Schlenk tube with a stir bar was affixed to a Schlenk line and flame-dried under vacuum. The flask was then evacuated and refilled 3 times with argon. In an air-free manner, the flask was then charged with 1,4-diethynyl benzene (126.2 mg, 1.0 mmol), 1,1′-diiodoferrocene (437.8 mg, 1.0 mmol), tetrakis-triphenylphosphine palladium 0 (46.2 mg, 0.04 mmol), copper­(I) iodide (7.6 mg, 0.04 mol equiv), diisopropylamine (0.167 mL, 1.2 mmol), and anhydrous dichloromethane (22.5 mL). The tube was also charged with 4-*tert*-butylphenylacetylene (chain stopper). The amount required depends on the desired size of the polymer chain. The Carothers equation was employed to determine the exact amount of chain stopper required (see Supporting Information).

After the addition of all reagents, the tube was capped with its stopper to form a closed system. The tube was disconnected from the Schlenk line. The contents were heated at 70 °C for 4 days with stirring at 900 rpm. After 4 days, the contents were allowed to cool to room temperature. The reaction mixture was then filtered through cotton, and the organics were washed with 5 × 20 mL of deionized water and dried over anhydrous sodium sulfate. The solvent was then slowly exchanged for hexane using a rotary evaporator. This was achieved by reducing the solution volume by approximately 25% using a rotary evaporator and reconstituting with a similar volume of hexane, after which the process is repeated until all the DCM is completely removed. During this process, a brown precipitate was formed.

The precipitate was then collected via centrifugation and washed immediately with 3 × 15 mL of hexane, 1 × 15 mL of diethyl ether, 5 × 15 mL of ethyl acetate, 2 × 15 mL of 1:1 dichloromethane/hexane, 3 × 15 mL of 7:3 dichloromethane/hexane, 3 × 15 mL of tetrahydrofuran, 1 × 15 mL of 8:2 chloroform/hexane, 1 × 15 mL of methanol, 3 × 15 mL of ethyl acetate, and 3 × 15 mL of hexane. The solids were then allowed to soak in 15 mL of hexane for no less than 12 h. The hexane was then removed by centrifugation, and the product was allowed to air-dry.

#### PIM-1 and Its oligomers

A clean 4 mL vial was charged with tetrafluorophthalonitrile (0.0600 g, 0.29 mmol), 3,3,3′,3′-tetramethyl-1,1′-spirobisindane-5,5′,6,6′-tetraol (0.1022 g, 0.29 mmol), potassium carbonate (0.3320 g, 2.4 mmol), anhydrous dimethylformamide (1 mL), and a stir bar. The vial was also charged with a freshly crushed catechol (chain stopper). The amount required depends on the size of the polymer chain desired. The Carothers equation was employed to determine the exact amount of chain stopper required (see Supporting Information). The vial was then capped and heated to 65 °C while stirring at 900 rpm for 3 days, after which the reaction mixture was poured into a minimum of 3 mL of deionized water. The yellow precipitate was then collected via vacuum filtration and washed twice with 5 mL of deionized water. The precipitate was then oven-dried for 1 h at 100 °C to afford the dry product as a yellow powder.

#### TBPIM-9 and Its Oligomers

A clean 25 mL one-neck round-bottom flask was charged with 2,5-dimethyl-1,4-phenylenediamine (0.2000 g, 1.47 mmol), dichloromethane (2 mL), and dimethoxymethane (1 mL, 11.30 mmol). The flask was also charged with *p*-toluidine (chain stopper). The Carothers equation was used to determine the exact amount of chain stopper required to synthesize polymer chains of a particular length (see Supporting Information). The contents were stirred and cooled in an ice bath, after which trifluoroacetic acid (4.5 mL, 58.77 mmol) was added in 3 portions over 20 min (1.5 mL/10 min; faster or slower additions were found to result in undesirable exotherms or short oligomeric product, respectively). The mixture was then stirred at 300 rpm for 48 h at room temperature (ca. 22 °C).

The mixture was then transferred to 30 mL of chilled aqueous ammonia and stirred for 22 h. The resulting off-white/brown precipitate was collected via vacuum filtration and washed with copious amounts of water until the pH was neutral. During this process, some oligomers formed a paste and were stuck to the walls of the flask and stir bar. This paste was washed with water until the pH was neutral, after which acetone (40 mL) was added to free the solids. Water (40 mL) was then added to the precipitate, and any oligomers dissolved in the acetone. Using the aforementioned filtration setup, the resulting precipitate was then collected. Solids were then washed with 40 mL of cold methanol (−20 °C) and allowed to air-dry on the funnel. The solids were then dried in a vacuum oven at 100 °C for 1 h to yield an off-white powder. The dried powder was then dissolved in a minimal amount of chloroform (ca. 5 mL) and reprecipitated in 11 mL of methanol. Solids were collected via vacuum filtration, washed with 10 mL of methanol, and allowed to air-dry.

## Supplementary Material



## Notes

1, 1′-Diiodoferrocene characterization spectra (^1^H NMR and HRMS); PIM-1 oligomer blending experiment procedure; Carothers’ equation calculations; effect of using experimental degrees of polymerization on sigmoidal fits (with related GPC data); Sigmoidal–Boltzmann fitting parameters; N_2_ isotherms for oligomers and polymers; and DBS data (PDF)^61–66^

## ⊥

R&D Process Department, NJ Biopharmaceuticals Inc., 08540, New Jersey, USA
