# Supplementary material for: Pore Network Percolation in Polymers of Intrinsic Microporosity Examined by Gaseous and Antimatter Probes
Source: Macromolecules. 2025 Oct 9;58(20):11171–9. doi: 10.1021/acs.macromol.5c00945 (PMC12573790; doi:10.1021/acs.macromol.5c00945)
Supplement: Supplementary file 1 [file ma5c00945_si_001.pdf]

Supporting Information for

Pore Network Percolation in Polymers of Intrinsic Microporosity (PIMs) Examined by Gaseous and Antimatter Probes

Darin Sukalingum,[1†] Tianran Zhai,[2†] Marc H. Weber,[3] Sohraab A. Khan,[1] Thant H. Htut,[1] and Jeremy I. Feldblyum[*1]

[1] Department of Chemistry, University of Albany, State University of New York, 12226, New York, USA.

[2] Department of Chemistry, University of Albany, State University of New York, 12226, New York, USA.
Current address: R&D Process Department, NJ Biopharmaceuticals Inc., 08540, New Jersey, USA

[3] Institute of Materials Research, Washington State University, Pullman, 99164, Washington, USA.

[†] These authors contributed equally to this work.

*Corresponding author email: jfeldblyum@albany.edu

Table of Contents

1. 1, 1'-diiodoferrocene characterization spectra 3
   - ^{1}H NMR spectrum 3
   - DART-MS (HRMS) spectrum 3

2. PIM-1 oligomer blending experiment procedure 4

3. Carothers' equation calculations 5

4. Effect of using experimental degrees of polymerization on sigmoidal fits 6
   - BET surface area vs. weight-average molecular weight (X_w) for PIM-1 6
   - GPC data for PIM-1 oligomers 7

5. Sigmoidal-Boltzmann fitting parameters 8

6. N_2 Isotherms 9
   - MPIM-1 oligomers and polymer 9
   - PIM-1 oligomers and polymer 10
   - TBPIM-9 oligomers and polymer 12
   - PIM-1 oligomer blending experiment 14
     - Physical blended mixtures 14
     - Solution blended mixtures 15
   - MPIM-1 Doppler broadening spectroscopy (DBS) samples 17

7. Doppler broadening spectroscopy data 18
   - Additional notes 18
   - Additional details on sample preparation and analytical methods 19
   - Doppler broadening spectroscopy data 21

8. References 24

1. 1, 1′–Diiodoferrocene characterization

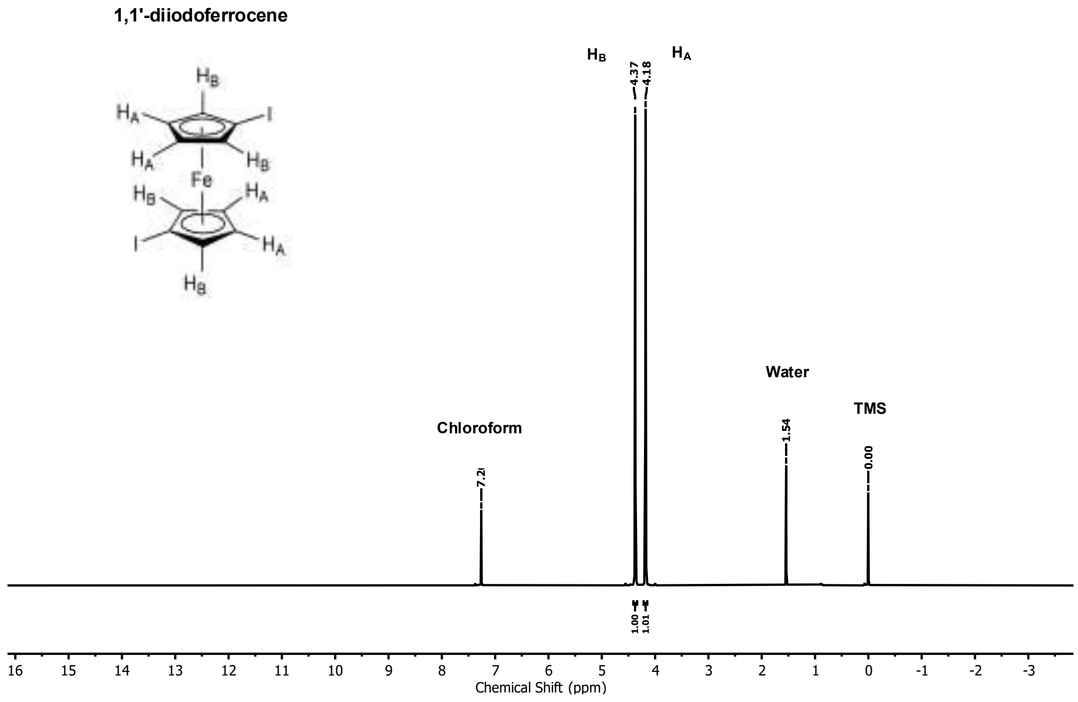

Figure S1: *^{1}H NMR (500MHz) of product (1, 1′-diiodoferrocene in $CDCl_3$)*

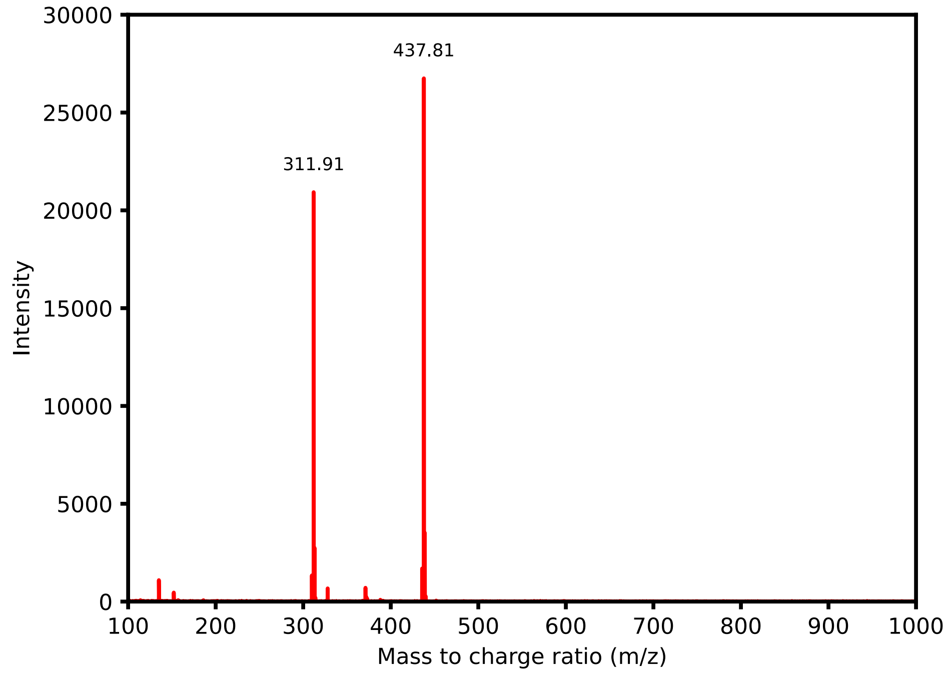

Figure S2: *DART mass spectrum of 1, 1′-diiodoferrocene. 437.8 (FcI_2^+), 311.9 (FcI^+)*

2. PIM-1 oligomer blending experiment procedure

To prepare a physically blended sample, dried portions of 90-mer and 5-mer of PIM-1 were weighed in appropriate proportions and mixed by sequential addition to the sample tube. Mixtures of 100 mg were prepared in this manner. Samples were then activated (as described in the main text) followed by collection of isotherm data for BET surface area determination.

Solution-blended samples were prepared by redissolving the physical mixtures described above in a minimal amount of THF (ca. 1 mL). This concentrated solution was rapidly transferred to deionized water (a minimum of 3 times the volume of the THF solution, ca. 3 mL) to yield a yellow precipitate. Solids were collected by vacuum filtration, washed with copious amounts of water, and dried at 100 °C in air. The dried sample was activated (as described in the main text) and its BET surface area measured.

We note that in preparation of the solution-blended samples, the use of any excess of THF for dissolution prevented effective precipitation in the subsequent step.

3. <u>Carothers' equation calculations</u>

As discussed in the main text, controlling pore system formation in polymers can be achieved by controlling M_n. To determine the amount of chain stopper needed to obtain polymers of targeted degrees of polymerization, a modified Carothers' equation for step growth polymerization was used (Equations S1 and S2).[1]

$$X_n = \frac{2}{2 - pf_{avg}} \tag{S1}$$

Equation S1 is the formula for the average degree of polymerization,[1] X_n. The variable p, in this context, is the percent conversion, which is assumed to be 100% (thus $p = 1$). Based on the degree of polymerization desired, one can solve for the average functionality (f_{avg}),[1] where:

$$f_{avg} = \frac{k_a N_a + k_b N_b + k_c N_c}{N_a + N_b + N_c} \tag{S2}$$

In equation S2, k represents monomer functionality, and N represents the number of moles or mole equivalents of that monomer. The subscript c corresponds to the chain stopper, whereas subscripts a and b correspond to the two (bifunctional) monomers. The molar equivalent of chain stopper (N_c) desired for a particular degree of polymerization (equation S3) is obtained by rearranging equation S2:

$$N_c = \frac{k_a N_a + k_b N_b - N_a f_{avg} - N_b f_{avg}}{f_{avg} - k_c} \tag{S3}$$

For TBPIM-9 polymerization, since there is only one monomer, equation S3 can be modified by removing all terms related to monomer b.

4. **Effect of using experimental degrees of polymerization on sigmoidal fits**

- BET surface area vs. weight-average molecular weight (X_w) for PIM

The solubility of PIM-1 and its oligomers allowed direct comparison of GPC-derived degrees of polymerization and BET surface areas (Figure S3).

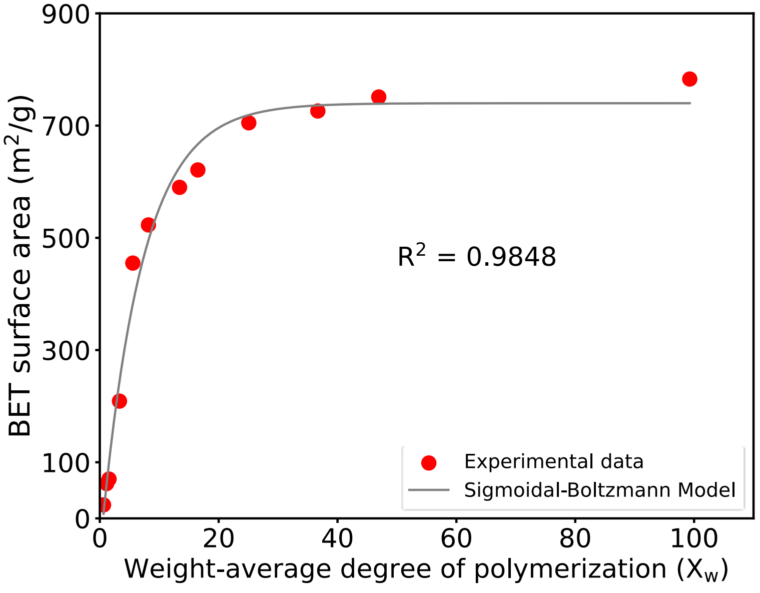

Figure S3: *BET surface area vs. weight-average degree of polymerization (with Sigmoidal-Boltzmann fit) for PIM-1. The black trace is a Sigmoidal-Boltzmann fit to the data.*

The overall features between the two trends utilizing calculated (Figure 1b, main text) and experimentally (GPC-) derived (Figure S3) degrees of polymerization are in agreement. The dataset derived from utilizing experimental degrees of polymerization values can also be well fit to a Sigmoidal model as seen in Figure S3. As such, regardless of whether calculated or experimental degrees of polymerization are utilized, the overall sigmoidal trend of the datasets are conserved. Discrepancies between the two trends can be attributed to either the polymerization reactions not approaching completion and/or structural incompatibilities between the polymer/oligomer samples and GPC calibration standards, both of which could account for the observed reduction in experimental chain lengths compared to the calculated values.

- GPC data for PIM-1 oligomers

Table S1: *Results from GPC analysis of PIM-1 oligomers and polymer.*

**Calculated X_w**	**GPC derived M_w**	**GPC derived X_w**	**Dispersity ($Đ_m$)**
3	621	0.6272	1.171
4	857	1.1464	1.305
5	1042	1.5534	1.413
10	1840	3.309	1.785
15	2862	5.5574	2.033
20	4056	8.1842	2.296
25	6437	13.4224	2.616
30	7836	16.5002	2.839
50	11731	25.0692	2.927
70	17013	36.6896	2.373
90	21671	46.9372	2.462
Polymer	45458	99.2686	3.688

5. Sigmoidal-Boltzmann fitting parameters

The equation of the Sigmoidal-Boltzmann fit can be expressed as,

$$y = A_2 + \frac{A_1 - A_2}{1 + e^{\frac{V_{50} - x}{Slope}}} \quad (S4)$$

A_1 and A_2 represent the initial and final values of the curve, V_{50} is the x value at y = 50% of the total maximum and "*slope*" refers to the steepness of the curve in the threshold region.

Table S2: *Sigmoidal-Boltzmann equation values used to generate fits to the datasets presented in the main text and SI.*

Dataset description	A_1	A_2	V_{50}	Slope
MPIM-1 BET surface area vs calculated degree of polymerization. (Figure 1a)	455.8161	3.2641	7.8279	0.4039
PIM-1 BET surface area vs calculated degree of polymerization. (Figure 1b)	727.5674	-561.4537	4.9651	9.2326
TBPIM-9 BET surface area vs calculated degree of polymerization. (Figure 1c)	577.0936	-79.8395	11.9443	4.1826
PIM-1 BET surface area vs weight-average degree of polymerization. (Figure S3)	739.9109	-1632204.3645	-52.4355	6.8823

6. **<u>N_2 Isotherms</u>**

- <u>MPIM-1</u>

Note: The isotherm associated with the MPIM-1 4-mer did not indicate measurable porosity and was therefore excluded. As such, this oligomer was assigned a BET surface area of 0 m^2/g.

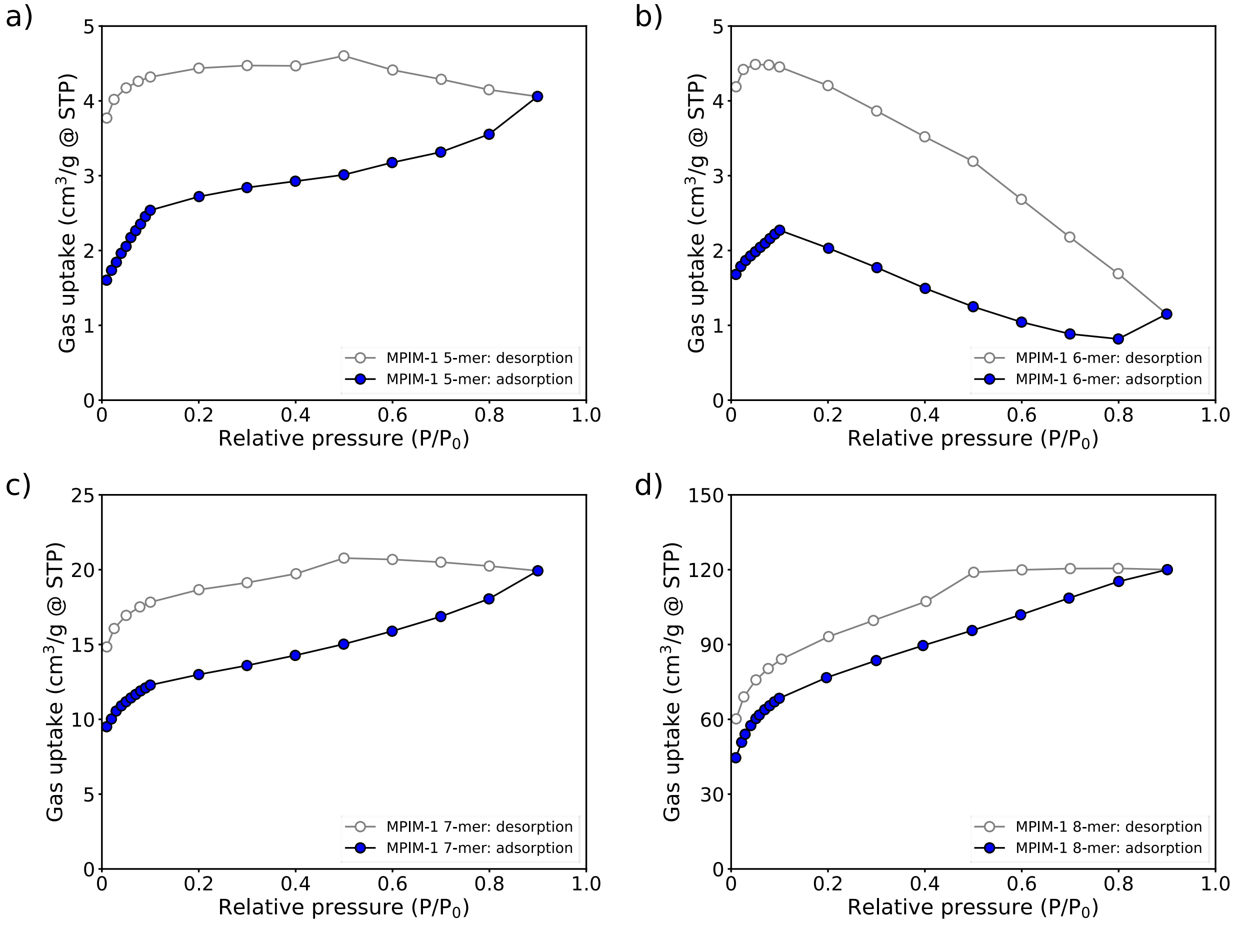

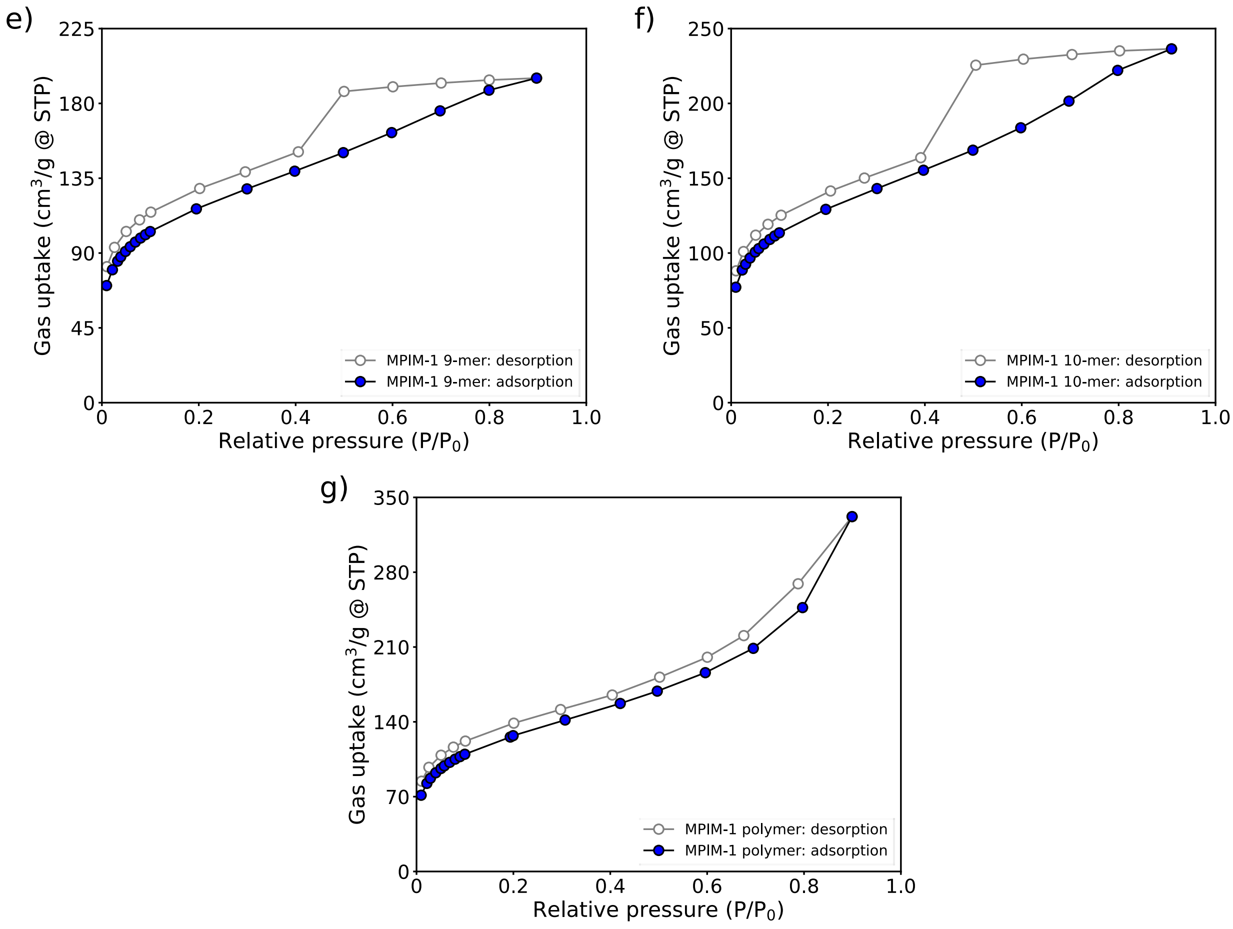

***Figure S4**: N_2 isotherm data for MPIM-1 a) 5-mer, b) 6-mer, c) 7-mer, d) 8-mer, e) 9-mer, f) 10-mer, and g) polymer.*

- <u>PIM-1</u>

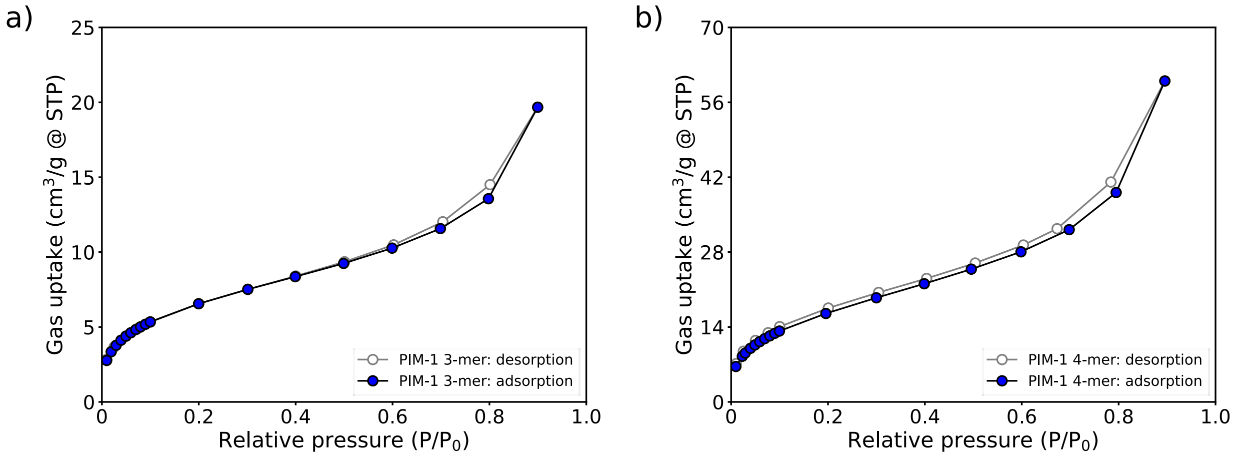

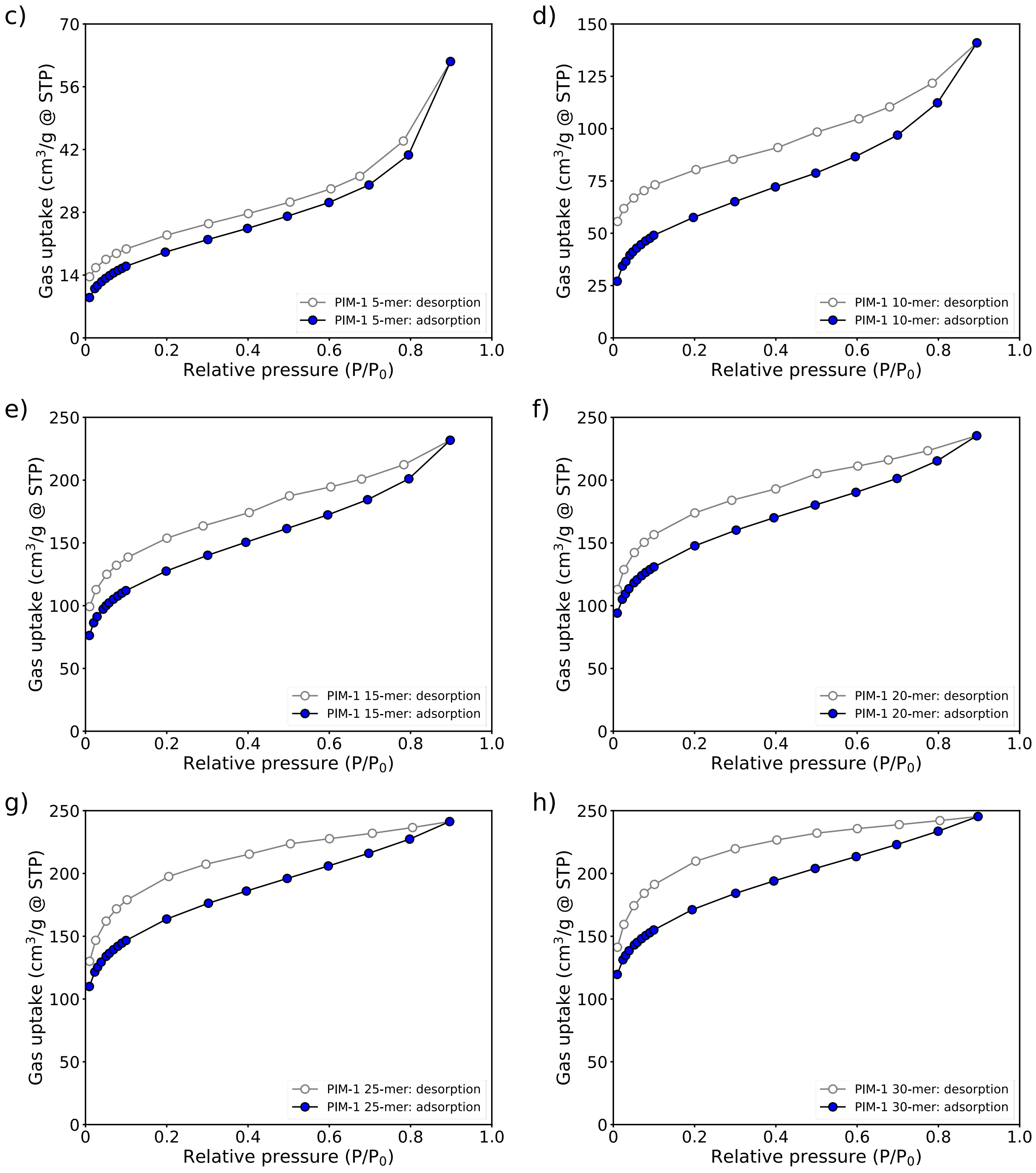
c)
d)
e)
f)
g)
h)
Gas uptake (cm^3/g @ STP)
Relative pressure (P/P_0)
PIM-1 5-mer: desorption
PIM-1 5-mer: adsorption
PIM-1 10-mer: desorption
PIM-1 10-mer: adsorption
PIM-1 15-mer: desorption
PIM-1 15-mer: adsorption
PIM-1 20-mer: desorption
PIM-1 20-mer: adsorption
PIM-1 25-mer: desorption
PIM-1 25-mer: adsorption
PIM-1 30-mer: desorption
PIM-1 30-mer: adsorption

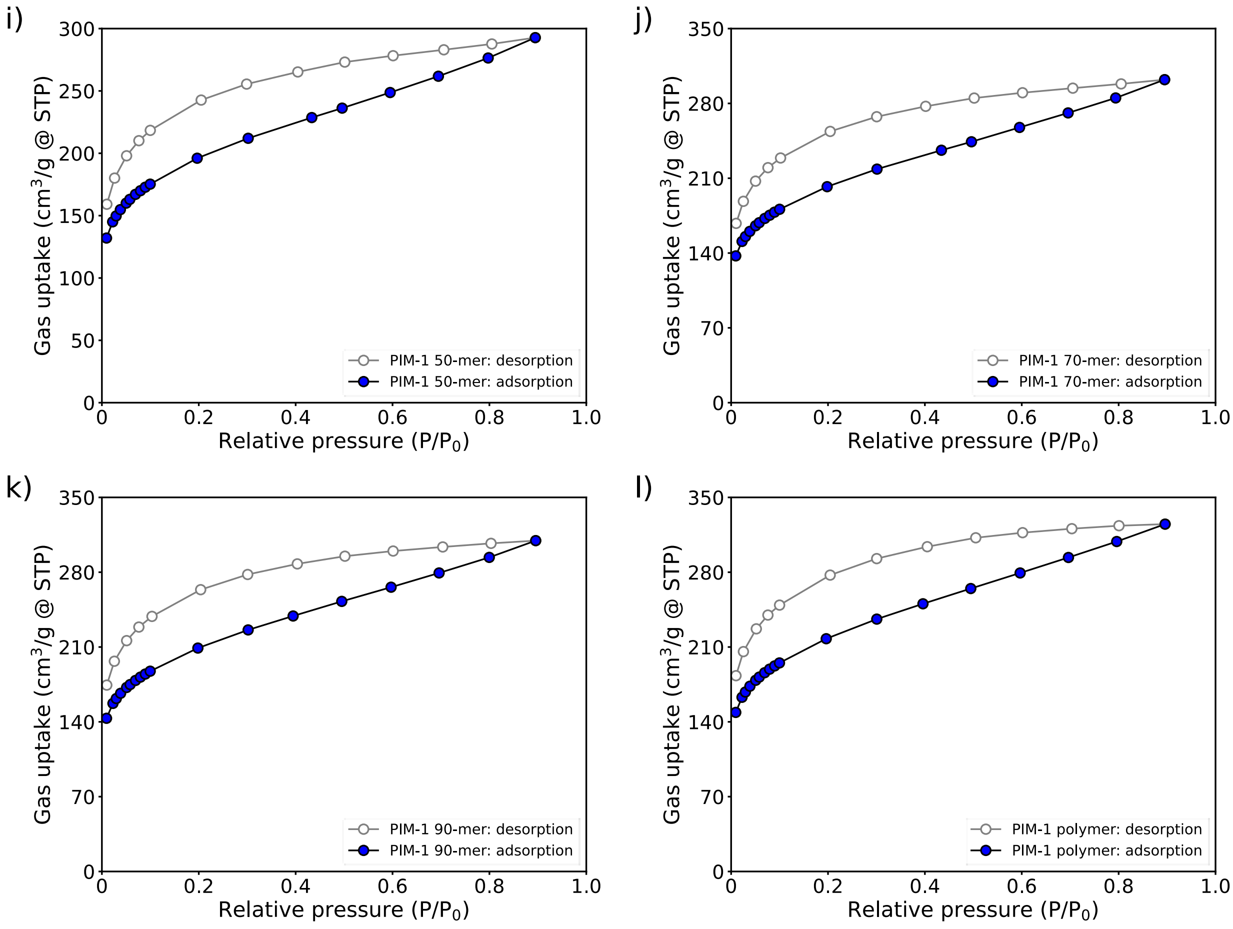

***Figure S5**: N_2 isotherm data for PIM-1 a) 3-mer, b) 4-mer, c) 5-mer, d) 10-mer, e) 15-mer, f) 20-mer, and g) 25-mer, h) 30-mer, i) 50-mer, j) 70-mer, k) 90-mer, and l) polymer.*

- <u>TBPIM-9</u>

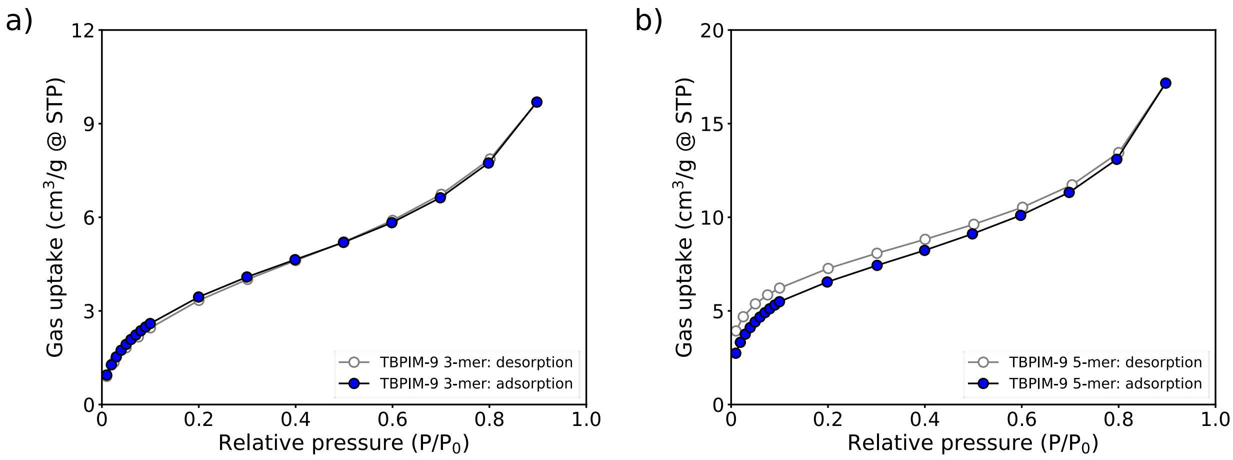

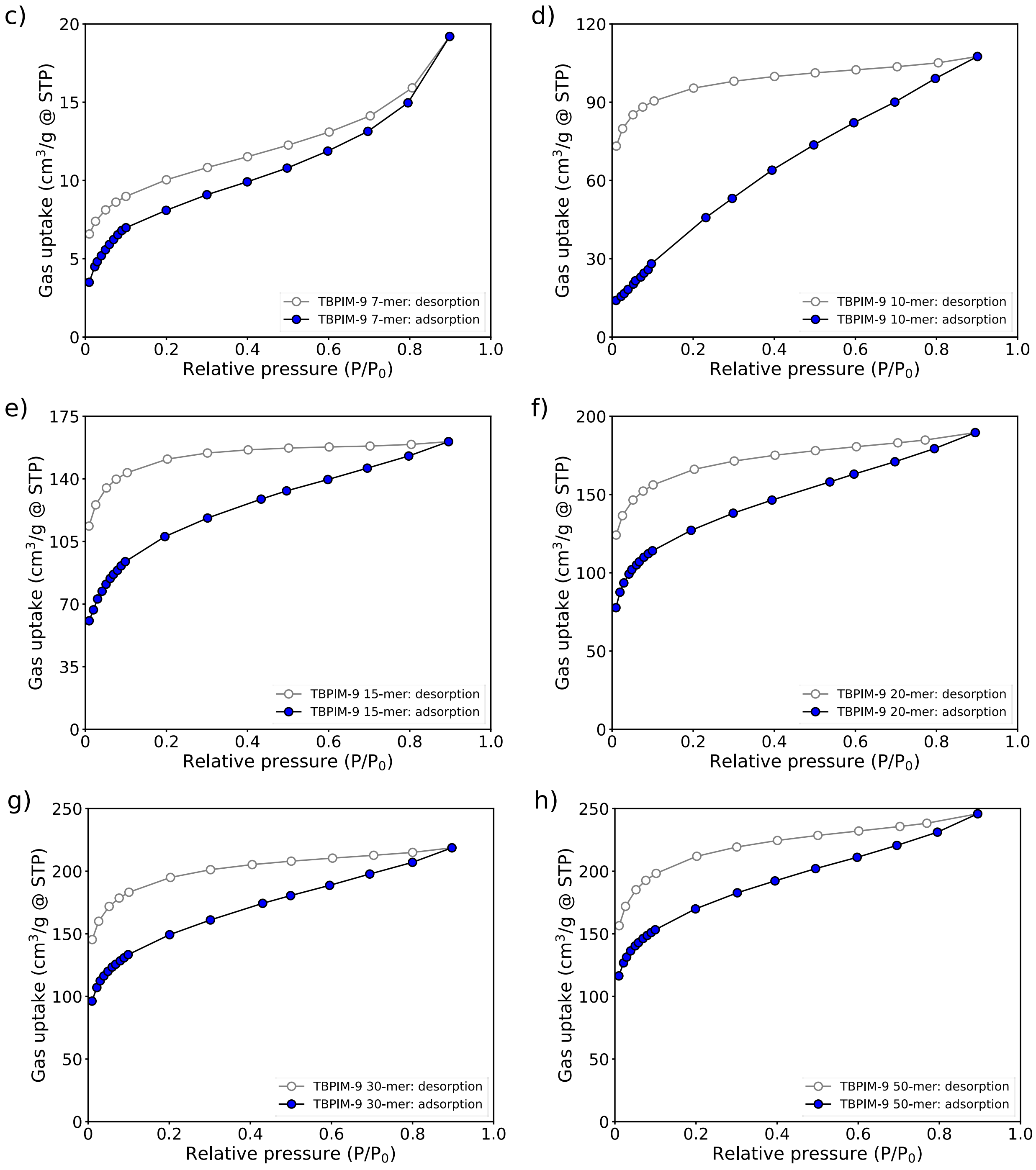
c)
Gas uptake (cm3/g @ STP)
Relative pressure (P/P0)
TBPIM-9 7-mer: desorption
TBPIM-9 7-mer: adsorption
d)
Gas uptake (cm3/g @ STP)
Relative pressure (P/P0)
TBPIM-9 10-mer: desorption
TBPIM-9 10-mer: adsorption
e)
Gas uptake (cm3/g @ STP)
Relative pressure (P/P0)
TBPIM-9 15-mer: desorption
TBPIM-9 15-mer: adsorption
f)
Gas uptake (cm3/g @ STP)
Relative pressure (P/P0)
TBPIM-9 20-mer: desorption
TBPIM-9 20-mer: adsorption
g)
Gas uptake (cm3/g @ STP)
Relative pressure (P/P0)
TBPIM-9 30-mer: desorption
TBPIM-9 30-mer: adsorption
h)
Gas uptake (cm3/g @ STP)
Relative pressure (P/P0)
TBPIM-9 50-mer: desorption
TBPIM-9 50-mer: adsorption

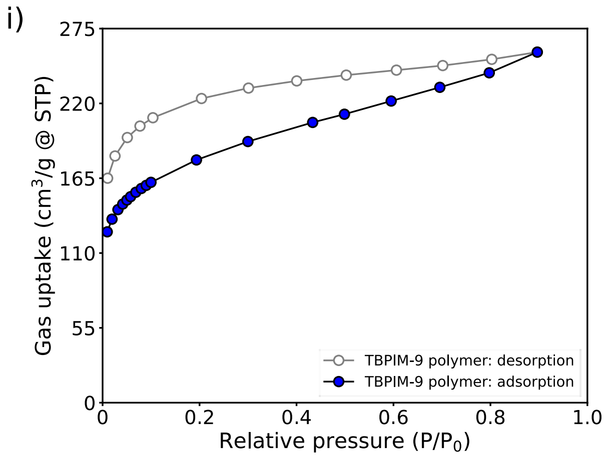

Figure S6: *N_2 isotherm data for TBPIM-9 a) 3-mer, b) 5-mer, c) 7-mer, d) 10-mer, e) 15-mer, f) 20-mer, g) 30-mer, h) 50-mer, and i) polymer.*

- <u>PIM-1 oligomer blending experiment</u>

  - <u>Physical blended mixtures</u>

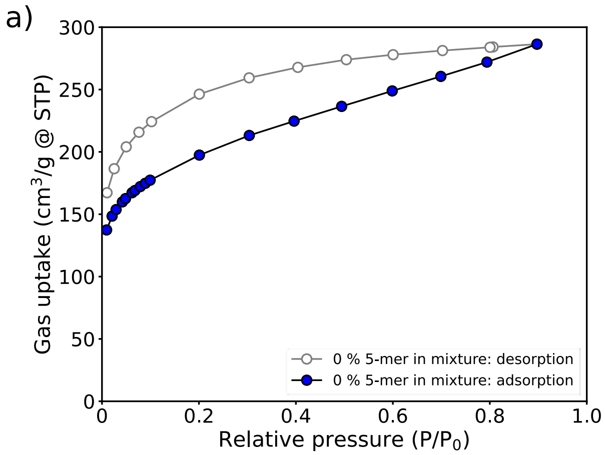

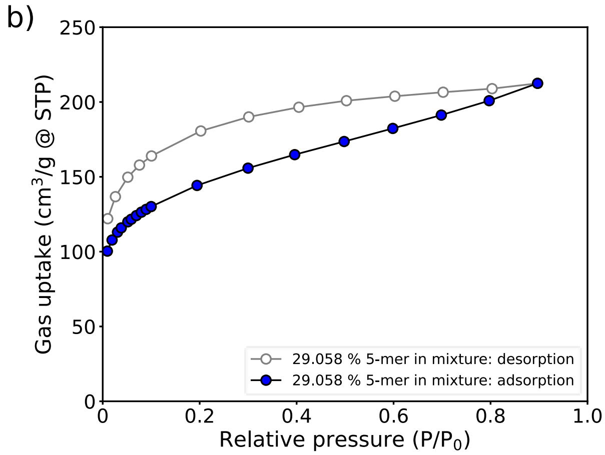

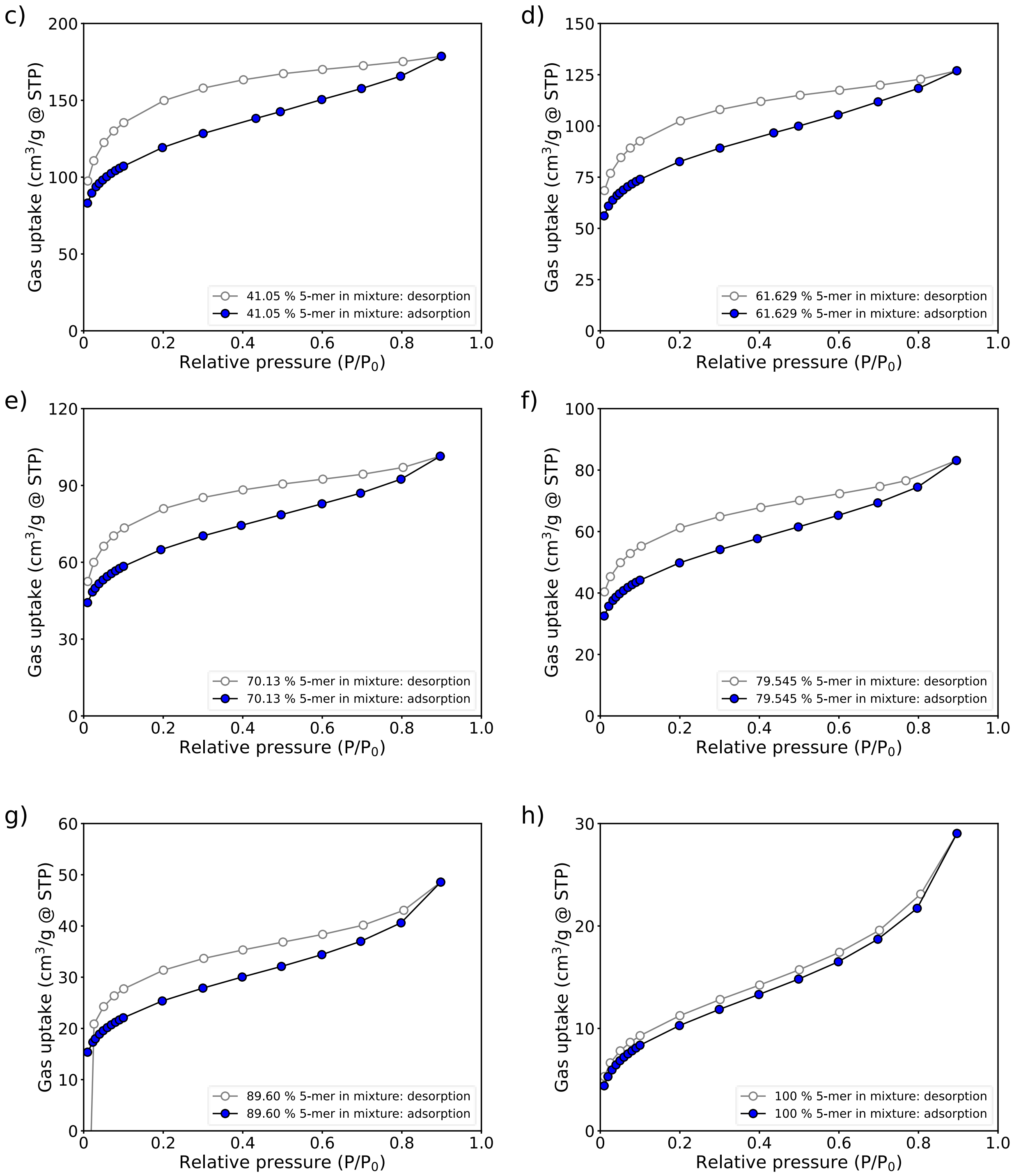

***Figure S7**: N_2 isotherm data for PIM-1 90-mer/5-mer physical mixtures containing a) 0 %, b) 29.058 %, c) 41.05 %, d) 61.629 %, e) 70.13 %, f) 79.545 %, g) 89.60 %, and h) 100 % 5-mer by mass.*

- <u>Solution blended mixtures</u>

Note: The accessible surface area associated with the PIM-1 100% 5-mer by mass solution blended mixture was too low to be accurately measured. As such, the associated isotherm was excluded.

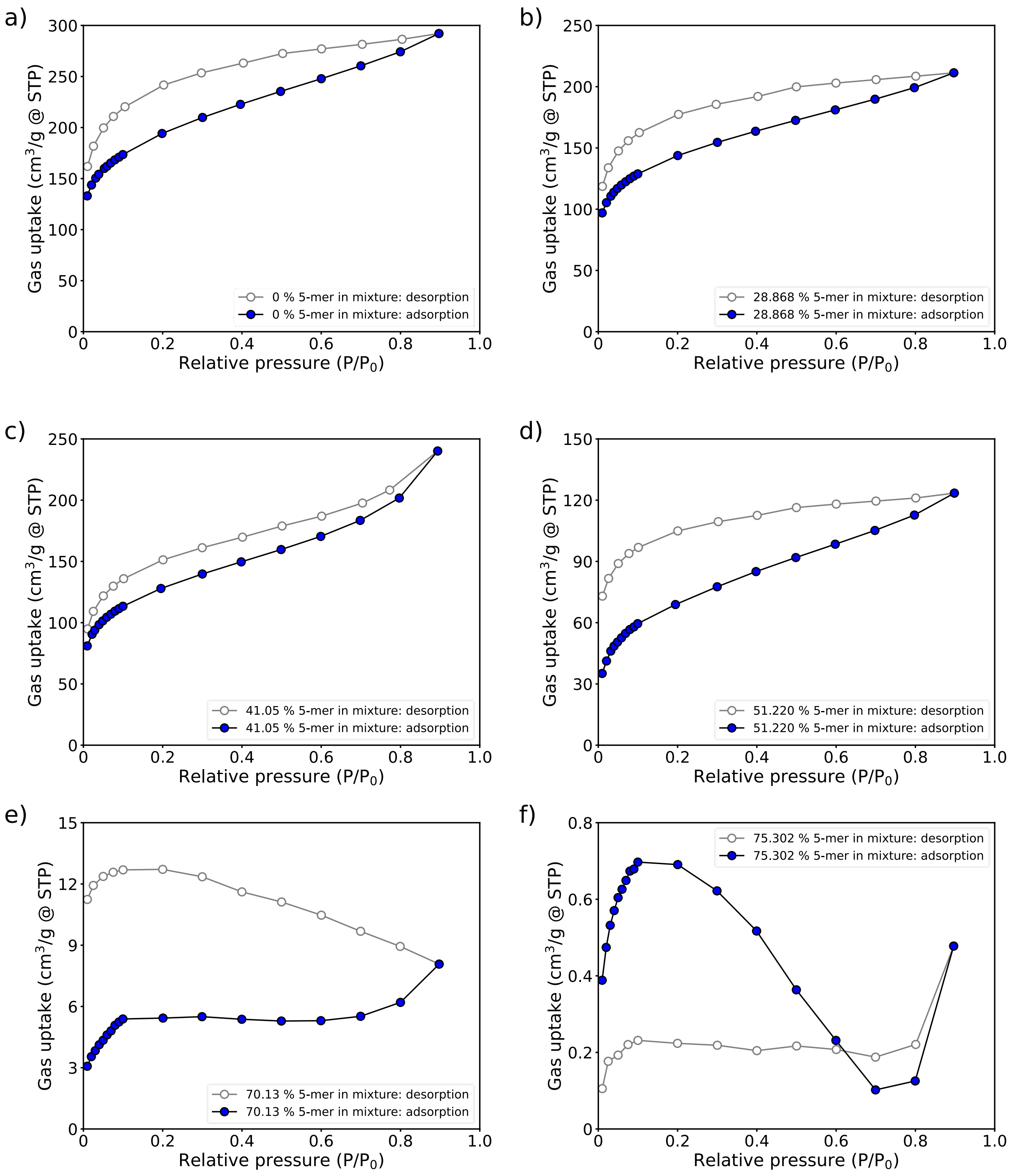

Figure S8: *N_2 isotherm data for PIM-1 90-mer/5-mer solution blended mixtures containing a) 0 %, b) 28.868 %, c) 41.05 %, d) 51.220 %, e) 70.13 %, and f) 75.302 %.*

- <u>MPIM-1 Doppler broadening spectroscopy (DBS) samples</u>

Note: The accessible surface area associated with the MPIM-1 5-mer (DBS) was too low to be accurately measured.

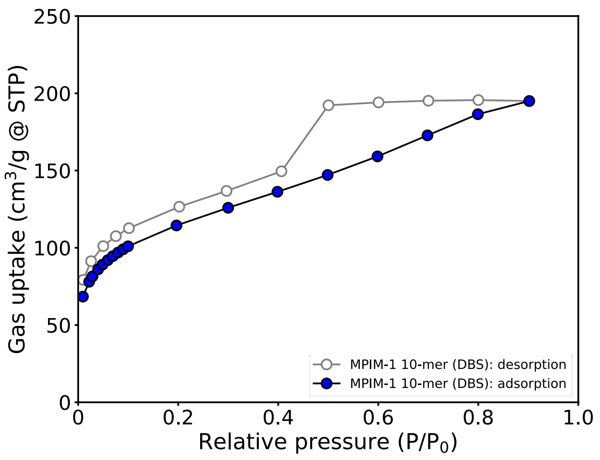

Figure S9: *N_2 isotherm data for MPIM-1 5-mer used in Doppler broadening spectroscopy (DBS) experiments.*

7. **Doppler broadening spectroscopy data**

- Additional notes

Positron/positronium doppler broadening spectroscopy (DBS) is a technique that can be used to detect atomic scale vacancies and open volume up to meso-porosity.[2, 3] As discussed in the main text, the sample is exposed to positrons of varying but controlled incident energy. Upon entering the sample, the positrons will thermalize (within 5 ps) and localize in nearby free volume or vacancies within the sample, followed by their eventual annihilation with electrons in the sample material. The incident positron energy can be converted to a mean implantation depth *d* following the empirical formula $d \cdot \rho = 40 nm \cdot g \cdot cm^{-3} \cdot (E/keV)^{1.6}$

In the host material or in small vacancy-sized open volume, 2 photon annihilations dominate. At the surface or in larger pores (≈ 1 nm and up) the positrons may bind to an electron to form a hydrogen like bound state called Positronium (Ps). Depending on the relative spin orientation two photon annihilations (opposite spins or annihilations with a second electron not bound to the positron) occur with Doppler shifted photon energies of $511 keV \pm \frac{1}{2} m_e p_p$ or annihilations into three photons (spin aligned) result in a broad energy spectrum up to 511 keV. Here 511 keV is the rest mass energy of the electron or positron and p_p is the momentum of the pair, dominated by the electron momentum.

The DBS analysis is based on the program 'Vepfit' that convolutes defect depth profiles with the implantation profile.[4] The defect profile consists of layers of constant annihilation parameters (S, W, or R), the positron diffusion length L and the respective layer thickness (as measured from the surface). Additionally, annihilations at the surface plane are treated separately. The depth profile is varied by Vepfit to minimize Chi^2. In the main text, the S and W

parameters are discussed extensively. The same cannot be said for the R parameter. The discussion of the R parameter from the main text continues here.

Typically, in fitting R assumes surface positronium formation only and no subsurface porous layers may exist in Vepfit. If R is fitted separately, three-photon annihilations due to porous layers can be included. A well-known porosity reference is required to relate unit-less R values to absolute porosities. In layer of Methyl Silsesquioxane (MSSQ) the open network resulted in a small porosity signal of 0.075 ± 0.002.[5] Adding 10% porosity raised the R value to 0.132 ± 0.005. Comparing these values to other materials may not be possible as the R value also depends on the properties of the pore walls, i.e. the host material.

Fitting the R data as a surface signal only results in significantly worse quality fits (Chi^2 doubles) than including the option of porous layers. A layer inclusive fit reveals a thin 0.57 ± 0.07 μm layer with some porosity (0.01082 ± 0.00052) for the MPIM-1 10-mer sample and 5-times less (0.00211 ± 0.00039) in the MPIM-1 5-mer sample. Down to a depth of 2.2 ± 0.4μm in the 10mer sample some residual porosity may be present (0.00433 ± 0.0007). Both values are small. If these 5-mer and 10-mer polymers can be scaled with the MSSQ data, less than 1% porosity resides in the 10mer sample top 0.57μm.

- <u>Additional details on sample preparation and analytical methods</u>

<u>Sample preparation</u>: Sample preparation protocol employed in our positron experiments are comparable to those described by Ming Liu *et al.*[6] The powders were received as coarse grains of (visually) sub mm particles. They were sprinkled onto double sided ¼ inch wide carbon tape and lightly tapped down. The tape was mounted between two bars about 1 inch apart. The bars and tape are mounted into the beam in a vertical position. The positron beam is aligned horizontally. Upon visual inspection, the polymer films form a solid covering layer on the carbon tape. To check for the risk of the particles falling off during the measurements under vacuum, additional data were collected at different times with no changes observed. At the end of the measurements the samples were removed from vacuum and inspected visually. No changes were observed.

The positron beam is aligned horizontally similar to the apparatus used in the Roberto Bursa group by Janusz D. Fidelus and coworkers in 2010.[7] In contrast to Ming Liu *et al.*,[6] monoenergetic positrons with variable energy are used to implant them at a specific depth below the surface. While Fidelus *et al.* used a pellet compressed under higher pressure, here the powder particles are loosely stacked together. Two detectors are used to pick up annihilation photons. They are mounted on opposite sides of the sample with the direction normal to the sample surface perpendicular to the line between the detectors and pointing into the positron beam. This geometry enables the coincident detection of both annihilation photons. A systematic challenge stems from small angle scattering of annihilation photons in the sample plane. In low-Z sample materials, some positrons backscatter and may reach and annihilate in the vacuum chamber wall steel. This effect is small in polymers and further suppressed by using only coincident events from both detectors. That greatly reduces the volume from where annihilations are observed and

eliminates the detection of annihilations from the vacuum chamber. In the more common single detector configuration, positrons enter the sample on one side and annihilation photons detected on the far side of the sample. The chance of small angle scattering is greatly reduced. If the diameter of the vacuum chamber around the incident positron beam is large (greater than 2 inches in the setup here), backscatter is suppressed.

Positron implantation: The positrons were implanted with incident kinetic energies from 20 electron volts (eV) up to 70 keV. A density of 1 g/cm^3 was assumed for the polymers. At the lowest energies below 100 eV, the positrons probe the top 1 nm of a sample. Here the samples are coarse grains with diameters on the order of micrometers. Locally, the positron will probe the top 1 nm of the surface of any grain near the top of the sample layer. At 400 eV, the top 10 nm are probed and at 7 keV the mean depth is 1 µm. This is the depth range where differences were observed between the samples. At higher implantation energies the data of the two samples investigated merged.

Local perspective: Compared to the grain sizes of the powder the mean implantation depth where differences are observed between the sample, the positrons probe thin layers of less than 1 µm, far less than the typical size of each grain. From the perspective of positrons implanted into the material this could be considered as probing a flat surface for each positron. Once the beam energy is sufficiently large to probe more than 2 µm into a material, some positrons may annihilate deep in a grain and others contribute from regions between the individual grains. At the highest energy, most likely the observed Doppler values are a geometric average of the grains and grain boundaries. Bear in mind that non-beam measurements involve positrons with energies up to 500 keV which may penetrate about a millimeter of material and even more so, generate a geometric average of grains, inter-grain regions, local surfaces and from deep in grains.

R-signal: The positronium R signal is shown for the top several 100 nm and also should reflect the surface of a single flat sample. These values are not calibrated with references for no and 100% Ps formation. Hence, no absolute scale is used. Using a Ge crystal at different thickness, (as used by Fidelus *et al.*[7]) density and temperature introduces too many systematic effects to be used for a reliable calibration.

- <u>Doppler broadening spectroscopy data</u>

Table S3: *S-parameter analysis for 5-mer and 10-mer of MPIM-1.*

Layer in sample	S parameter (S)	Absolute uncertainty (±) of S	S parameter for bulk (S_{bulk})	Absolute uncertainty (±) of S_{bulk}
5-mer surface	0.48106	0.000548	0.48199	0.00019
5-mer (<1.27 ± 0.26 um)	0.4803	0.000225	0.48199	0.00019
5-mer bulk	0.48209	0.0002	0.48199	0.00019
10-mer surface	0.48356	0.000398	0.48199	0.00019
10-mer (<1.6 ± -- um)	0.4811	0.00037	0.48199	0.00019
10-mer bulk	0.48188	0.000317	0.48199	0.00019

Table S4: *W-parameter analysis for 5-mer and 10-mer of MPIM-1.*

Layer in sample	W parameter (W)	Absolute uncertainty (±) of W	W parameter for bulk (W_{bulk})	Absolute uncertainty (±) of W_{bulk}
5-mer surface	0.04652	0.000152	0.04572	0.00005
5-mer (<1.27 ± 0.26 um)	0.04647	0.000059	0.04572	0.00005
5-mer bulk	0.04567	0.000051	0.04572	0.00005
10-mer surface	0.04565	0.000102	0.04572	0.00005
10-mer (<1.6 ± -- um)	0.04568	0.000096	0.04572	0.00005
10-mer bulk	0.04577	0.000082	0.04572	0.00005

Table S5: *S/S_{bulk} and W/W_{bulk} values for 5-mer and 10-mer of MPIM-1.*

Layer in sample	S/S_{bulk}	Absolute uncertainty S/S-bulk	W/W_{bulk}	Absolute uncertainty W/W-bulk
5-mer surface	0.99807	0.00120	1.01750	0.003506
5-mer (<1.27 ± 0.26 um)	0.99649	0.00061	1.01640	0.001703
5-mer bulk	1.00021	0.00057	0.99891	0.001561
10-mer surface	1.00326	0.00092	0.99847	0.002484
10-mer (<1.6 ± -- um)	0.99815	0.00086	0.99913	0.002367
10-mer bulk	0.99977	0.00077	1.00109	0.002101

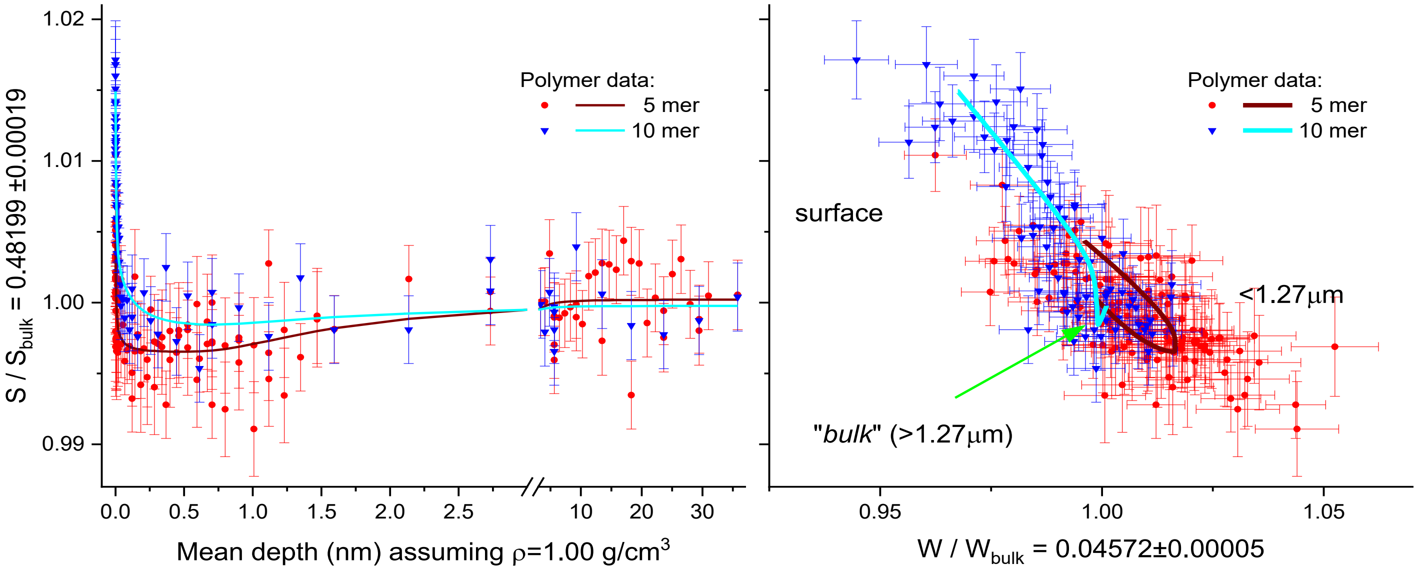

Figure S10: *S/S_{bulk} vs. mean depth (left) and W/W_{bulk} (right) for the 5- and 10-mer oligomers of MPIM-1.*

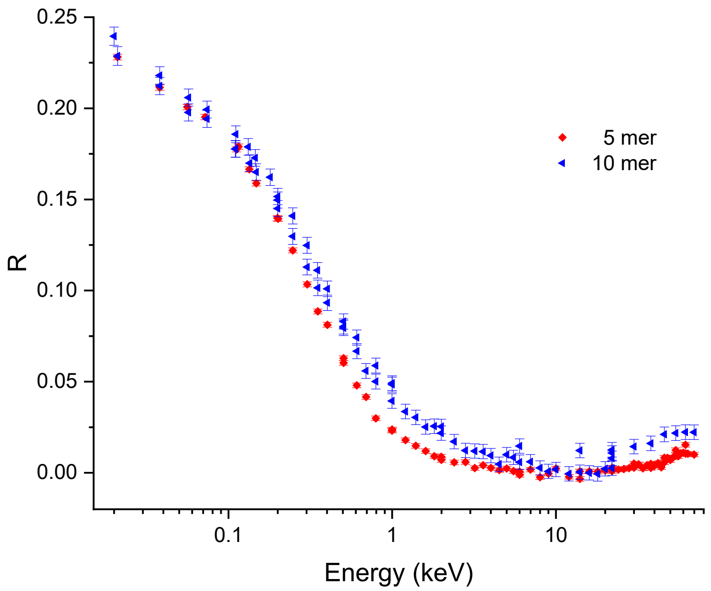

Figure S11: *R-parameter vs. full positron energy (eV) range for the 5- and 10-mer oligomers of MPIM-1. Above 10 to 20 keV, systematic effects due to sample orientation and thickness cause changes.*

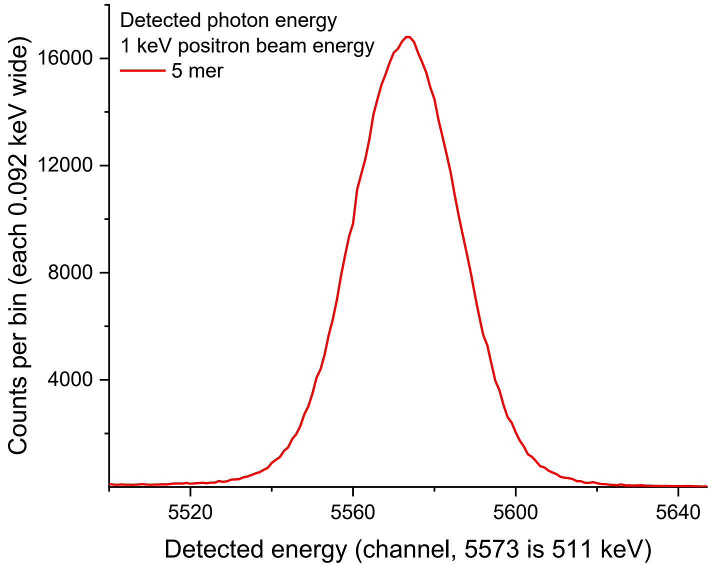

Figure S12: *DBS annihilation line (Counts vs. energy) for the 5-mer of MPIM-1 (photon-annihilation photoelectric peak region only).*

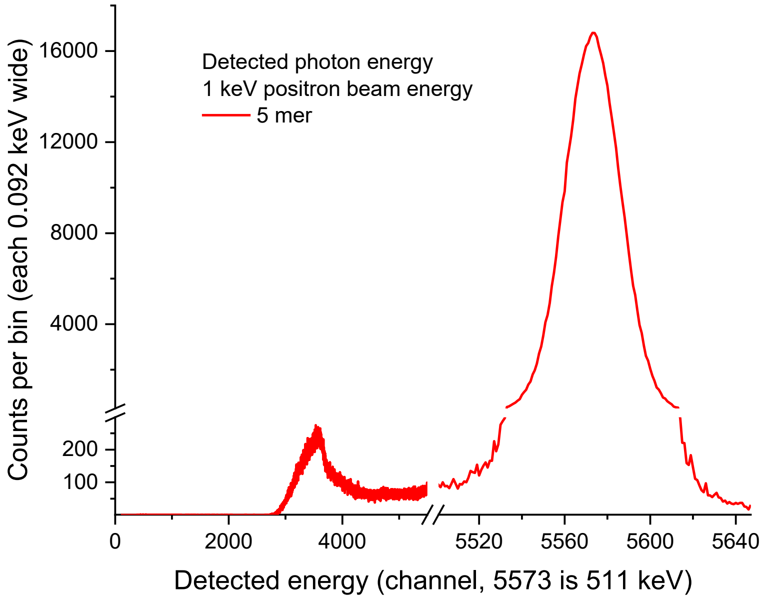

Figure S13: *DBS annihilation line (Counts vs. energy) for the 5-mer of MPIM-1 (Full energy and count ranges). Compton scattering edge (channel 3685) and three photon annihilation region (valley, channel 4000 to 5500) becomes visible.*

8. References

(1) Odian, G. *Principles of Polymerization*; McGraw-Hill, 1970. DOI: 10.1002/047147875X.

(2) Schultz, J. P.; Lynn, G. K. Interaction of positron beams with surfaces, thin films, and interfaces. *Reviews of Modern Physics* **1988**, *60* (3), 701-779. DOI: 10.1103/RevModPhys.60.701.

(3) Gidley, W. D.; Peng, H.-G.; Vallery, S. R. Positron annihilation as a method to characterize porous materials. *Annual Review of Materials Research* **2006**, *36* (1), 49-79. DOI: 10.1146/annurev.matsci.36.111904.135144.

(4) Veen, V. A.; Schut, H.; Clement, M.; Nijs, D. J. M. M.; Kruseman, A.; Ijpma, M. R. VEPFIT applied to depth profiling problems. *Applied Surface Science* **1995**, *85*, 216-224. DOI: 10.1016/0169-4332(94)00334-3.

(5) Petkov, M. P.; Wang, C. L.; Weber, M. H.; Lynn, K. G.; Rodbell, K. P. Positron Annihilation Techniques Suited for Porosity Characterization of Thin Films. *The Journal of Physical Chemistry B* **2003**, *107* (12), 2725-2734. DOI: 10.1021/jp013947b.

(6) Liu, M.; Wong-Foy, G. A.; Vallery, S. R.; Frieze, E. W.; Schnobrich, K. J.; Gidley, W. D.; Matzger, J. A. Evolution of Nanoscale Pore Structure in Coordination Polymers During Thermal and Chemical Exposure Revealed by Positron Annihilation. *Advanced Materials* **2010**, *22* (14), 1598-1601. DOI: 10.1002/adma.200903618.

(7) Fidelus, J. D.; Karbowski, A.; Mariazzi, S.; Brusa, R. S.; Karwasz, G. Positron-annihilation and photoluminescence studies of nanostructured ZrO2. *Nukleonika* **2010**, *55* (1), 85-89.
